# Unending dialectical politics of identity in Ethiopia

**DOI:** 10.12688/f1000research.160666.1

**Published:** 2025-01-27

**Authors:** Tefera Assefa

**Affiliations:** 1 Center for Federalism and Governance Studies, Addis Ababa University College of Law and Governance Studies, Addis Ababa, 1176, Ethiopia; 2 Public Administration and Development Management, Ambo University, Woliso, Oromia, Ethiopia

**Keywords:** Ethiopia, Pan-ethnic nationalism, Pan-Ethiopian nationalism, Identity politics, unending dialectical

## Abstract

**Background:**

Identity politics is one of political terminology which is subjected to continuous and increasingly contested conceptualization and use. Moreover, identity exists in all social level starting for individual to international level, which makes identity a multi-layer concept. There has been a great deal of identity-based terminological distortion and misconception in Ethiopian political discourses and practice since its formation in the modern form. One of the primary challenges in Ethiopian present political discourse is misconception, complexity, and contradiction between ethnic identity (የብሄር ማንነት) and civic identity or citizenship (የዜግነት ማንነት), which must be addressed in Ethiopian political discourse.

**Methods:**

In this paper, critical post-modernism research philosophy and Dialectical Method is used to analyze the rotation of contradiction between Pan-Ethiopianism and Pan-ethnic nationalism. The narrative analysis is used to make a critical investigation of the vicious cycle unending dialectics of identity politics in Ethiopia.

**Discussion:**

The current political situation is an indication of unending dialectics of Ethiopian politics. The existing politics of ethnic identity and citizenship indicate unending dialectic of political friction with the rotation of thesis and anti-thesis which left the state building projection of country incomplete and weak dialectical process. Wrong policy and political responses of successive regime to the nationality questions (quests for power sharing and autonomous self-government) resulted in the sustained and vicious circle of conflict.

**Conclusion:**

Generally, it is argued that prescription of one side of nationalism at the expense of the other will never resolve the political crisis of the country. Therefore, it is recommended that both integrationist and accommodationist institutional design and multinational democratic federalism should be implemented effectively to cope with the salience of identity politics in the country.

## 1. Introduction

Identity politics is political terminology that is subjected to continuous and increasingly contested conceptualization and use, mostly in developing countries. Moreover, identity exists at all social levels, starting at the individual to international level, which makes identity a multi-layer concept in which multiple identities coexist at each level and the salience of any particular identity ebbs and flows depending upon the circumstances ( Phillips, 2016; Muir & Wetherell, 2010). In modern political theory, identity politics is an integral part of both the conflict and peacebuilding processes ( Orjuela, 2008; Heywood, 2019). More importantly, identity politics are also used as an instrument of nation or state building most of the time used by the dominant group ( Leach, Brown & Worden, 2008). However, most of the time, the role of identity and its political manipulation by dominant groups is not considered as identity politics, and it is mistakenly linked to and equated with the politics of minority groups that look for liberation from social, political, and economic repression ( Holberg Prize, 2021).

Along with strong identity-based contestation, there have been many identity-based terminological distortions and misconceptions in Ethiopian political discourse and practice since its formation in the modern form. In Ethiopian political discourse, there is uncertainty over ethnic identity racism, and ethnic prejudice. Any definition of racism cannot encompass Ethiopia’s past and present quests for ethnic identity (identity politics). According to various definitions of racism, the quest for ethnic identity recognition and self-determination in Ethiopia in the past and now cannot simply be equated with racism and ethnic prejudice. However, the extremist political elite (the pseudo pan-Ethiopian nationalists for instance) consider the quest for alternative national identities as racism ( ዘረኝነት ). Furthermore, in Ethiopian political discourse, there are persistent tensions between nationality and citizenship. Treating these two phrases as synonyms is a risky political calculation, especially in multinational and multicultural countries.

According to theoretical and empirical data, the link between ethnicity (nation/nationalism) and citizenship is complicated and complex ( Sant, Davies & Santisteban, 2016; Hristova & Cekik, 2013). Nationalism and citizenship are used interchangeably. However, in both theory and practice, these two concepts are distinct. Several scholars have discussed the relationship between nationalism and citizenship. Some academics correlate nationalism with citizenship, whereas others seek to establish a relationship between the two categories. Others draw a clear contrast between the two terms, while others associate nations with the modern nation-state, in which nation and state coexist/coincided ( Piattoeva, 2016; Kaufman & Williams, 2017; Indian Today, 2018). “A lot of the confusion stems from the fact that ‘citizenship’ is usually confounded with ‘nationality/nationalism’, and sometimes the two, conceptually different, terms, are regarded as identical and used interchangeably” ( Meehan 1997 cited in Tsaliki, 2007, p. 162) and nation-building has been used by Jacobin unitarists to build nation-state from multinational community ( McGarry and O’Leary, 2009 and 2005). However, the two terms are significantly different from each other. Particularly in multinational or multicultural countries, it is a dangerous political calculation to treat the two terms as synonyms. In particular, with the advent of the multinational modern constitutional state, the difference between the two terms became clear. To quote the words of Tsaliki (2007, p. 162), “at the dawn of the modern constitutional state, the two terms differed greatly.” Moreover, the old model of the nation-state project failed with the emergence of a quest for alternative identity, which led to the development of multiculturalism and multinational federalism ( Kymlicka, 2007).

In Ethiopia, nationalism and citizenship cannot be used interchangeably. This is due to the fact that Ethiopia could not be established or developed as a nation (nation-state). In Ethiopian history, there has been major elite-based tension between nationalism and citizenship, which stems from a poor definition and understanding of the two categories. Ethiopian political conflict of identity is defined by a vicious loop of contradiction between thesis and anti-thesis ( Assefa, 2022).

As a result, one of the primary political challenges in Ethiopian present political discourse and history is the misunderstanding, complexity, and contradiction between ethnic identity ( የብሄር ማንነት ) and civic identity or citizenship ( የዜግነት ማንነት ), which must be addressed in Ethiopian political discourse. On the one hand, the slogan of one nation, one language, one religion, and one identity remain visible, while on the other hand, there is a rising proclivity for ethnic-based identification. Lyons (2019, p. 3) clearly put that “Ethnic identity and Ethiopian identity have an extensive scholarship and are key parts of contemporary Ethiopian politics.” Such confusion should be critically dealt with to create an all-inclusive and mutually agreed upon democratic citizenship in which both identities are truly recognized rather than building one at the expense of others. Hence, this article aims to critically analyze the unending dialectical contradiction between pan-Ethiopian and pan-ethnic nationalism.

## 2. Methods

In this article, secondary sources of data, such as books, articles, working papers, reports, videos, audios, and interviews held by different media, were used to indicate how the current narration is rooted in the imperial projection of nation-building. Narrative analysis is used to indicate how pan-Ethiopian nationalism has shifted to ethno-religious nationalism discourse held by politico-religious elites and the response to the narrations by the proponents of multi-cultural nationalism. Therefore, Dialectical critical post-modernism of research philosophy is used to analyze the rotation of the contradiction between Pan-Ethiopianism and Pan-ethnic nationalism. Hence, an attempt has been made to develop a dialectical model that shows the political discourse of the country since the 1991. Moreover, the pre-1991 dialectical model of Assefa (2022) and post-1991 dialectical models are combined to develop a comprehensive dialectical model that indicates the cyclical rotation of thesis and anti-thesis and the unending dialectic of Ethiopian identity politics, which results in a vicious circle of ethnic conflict in the country ( Assefa, 2022; Jima, 2021). The year 1991 was taken as a critical juncture that resulted in a complete shift of the post-1991 nationality/identity discourse and brought identity politics into the Ethiopian political history of the country.

## 3. The Post-1991 Political Discourse in Ethiopia

The political watchwords at the current time (post-2018) revolved around nation building, which emanated from the faulty definition of state and nation. Throughout the historical and current politics of Ethiopia, it is common to observe the misconceptions of nation-building and state-building. Nation-building discourse could not be a solution for the sequences of past political mistakes, current political problems, and for future state-building and peaceful coexistence in multinational states like Ethiopia. Ethiopia is a multinational state that has no single national identity but identities that should/need to be recognized, which have never been genuinely recognized throughout its history and in contemporary politics of the country. Here, the political headache of the country is not only a contradiction between the quest for such multinational identities and a single national identity, but also the ways in which the contestation is treated by successive governments ( Assefa, 2022; Gudina, 2011; Jalata, 2010 and 2020; Jima, 2021).

The rejection of the desire for a multinational identity by the ethno-religious elite and pseudo-Pan-Ethiopian nationalists exacerbated ethnic hatred and strife. Along with this, the country’s post-1991 politics failed to create a multinational federalism and to address nationality issues in meaningful ways. As a result, there is a plethora of assertions that ethnicity is controlled by the political elite and is utilized as a tool of divide and rule. It is thought that multinational federalism was used as a pretext by the TPLF elites to influence and mobilize the community for political and economic benefits by the EPRDF/TPLF. For example, one can easily imagine how the EPRDF government unconstitutionally responded to numerous ethnic groups’ constitutional quest for self-determination in the SNNPs region (the case of the Sidama People, Silte and Gerageh, Wolayita, etc.) and other parts of the country, implying a lack of genuine implementation of true federalism elsewhere in the country, resulting in violent ethnic conflict, numerous deaths, dislocation, and devastation ( Dessalegn & Afesha, 2019; Halabo, 2019 & Belay, 2013).

In addition to the aforementioned issues, there is a propensity to preserve the dominant culture, which is based on the pan-Ethiopian identity of the previous imperial regime. This has exacerbated the political division between pan-ethnic and pan-Ethiopian nationalism. In the country’s political discourse, so-called dominant groups attempted to maintain their dominance through ethnic closure. This could not go unopposed, resulting in the protests of the disadvantaged (or so they thought) ethnic groups toward the ethnic closure of the so-called dominant culture. This resulted in mutual ethnic polarization, as described in Ballard. For instance, Ballard (2002, p. 28) states that

The privileged groups routinely close ranks in ethnic terms to exclude their social subordinates in hegemonic patterns, so the excluded frequently respond by closing ranks themselves, the better to resist and subvert their subordination. When each side reacts in turn against the other, the outcome is often a rapid and escalating process of mutual ethnic polarization.

Such mutual ethnic polarization has been found in Ethiopian present and historical political debates. The current political squabbles and violent war in Ethiopia are unquestionably the product of such an identity-based political division between pan-Ethiopian and pan-ethnic nationalists ( Zerai, 2017). Furthermore, the division is reflected by the contentious interpretation and misuse of ethnic-based federalism hosted by the EPRDF, which remains a major political issue in the country.

There are widespread claims that ethnic/multinational federalism in Ethiopia, which granted (at least in principle) the right to self-determination, self-expression, and the protection and recognition of ethnic-based identities, has never been a source of political conflict in the country as it was claimed ( Assefa, 2022). Pan-ethnic nationalists said that the difficulty in this respect is the lack of actual implementation of true multinational federalism and the EPRDF government’s unlawful reaction to constitutional questions. It is also worth noting the contributions of proponents of Pan-Ethiopian nationalism, who argued that the EPRDF/TPLF uses federalism as a means of divide and rule in the country’s present political discussions. Yusuf (2019a, b) clearly stated the paradox of ethnic federalism in Ethiopia, stating that ethnic federalism simultaneously empowers and disempowers ethnic groups. The upshot of this paradox is that ethnic federalism grants autonomous power (in theory) to regional authorities and empowers formerly excluded ethnic groups on the one hand, while party tentacles exercise powerful impacts and rigorous control over government structure on the other hand ( Zerai, 2017; Yusus, 2019a & b; Marzagora, 2017). Moreover, the federal structure disempowered the intra-regional minority equally. Marzagora (2017, p. 26) states that

The state-sponsored nationalism in Ethiopia of today (EPRDF era) thus draws both from the Great Tradition and from counter-historiographies. It attempts to accommodate and selectively emphasize one or the other. The contradictions between the two remain, and the new national ideology promoted by the EPRDF does not in any way represent a synthesis between the nations, nationalities, and peoples on one side and the imperial heritage on the other.

This, in turn, hampers the effective implementation of true and democratic federalism throughout the country, which later resulted in widespread political opposition from both Pan-ethnic nationalists and Pan-Ethiopian nationalists, who then sandwiched and disintegrated the EPRDF ( Zerai, 2017; Yusus, 2019a & b).

**Figure 1.**
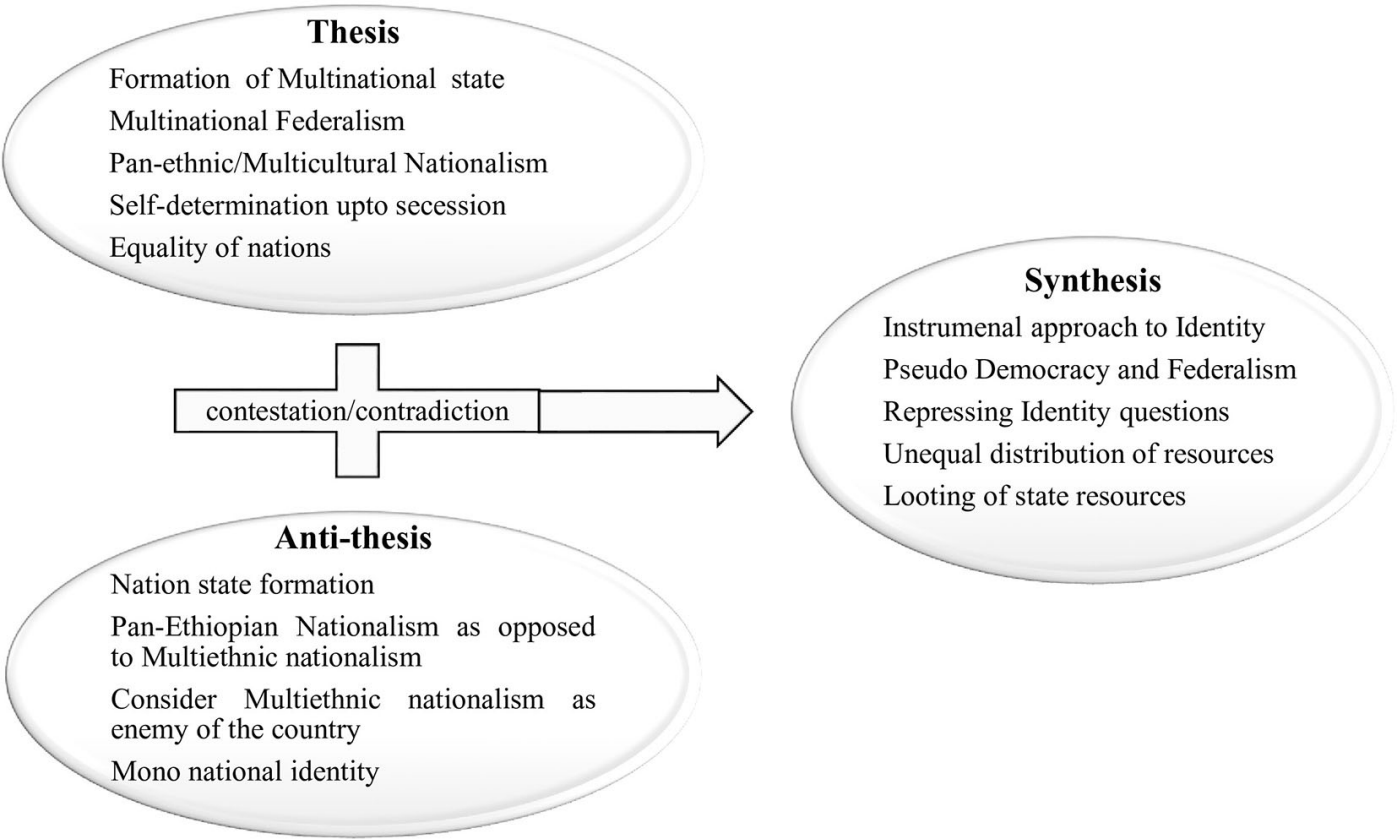
Dialectical model of Post-1991 Politics of Identity in Ethiopia. Note: Adapted from Assefa (2022) and supported by other literature (2024). The figure is adapted from previous publication which is free to adapt according the license policy of the journal license. Additionally, the previous figure is the design of the author.

Considering the dialectical process in light of this, there are still two contradicting and extreme questions regarding nationality and citizenship. This dialectical process is shown in dialectical triads as follows ( Figure 1). The dialectical process of Ethiopian political history is always incomplete, considering the triads of dialectical process, thesis, anti-thesis, and synthesis. The historical and contemporary politics of the country indicate a vicious cycle of contestation, which is characterized by unending friction between thesis and anti-thesis ( Assefa, 2022).

Based on the Hegelian dialectical triad shown in Figure 1, the post-1991 political contestation between competing nationalism can be represented by a dialectical model. The model was adopted from Assefa’s paper, “The Imperial Regimes as a Root of Current Ethnic-Based Conflicts in Ethiopia,” and it depicts dialectical contestation drive from the nation-state formation that took place pre-1991 ( Assefa, 2022). The reciprocal of Assefa’s model is used in this article to illustrate how the country’s post-1991 politics shifted from the imperial regime’s thesis to the anti-thesis, and vice versa. The pursuit of pan-ethnic nationalism has not been acknowledged by succeeding governments, according to Assefa’s model. During the 1960s and the 1970s, student revolution and aftermath, the quest for pan-ethnic nationalism attracted a wide range of individuals and political groups to challenge the narrow definition of Ethiopianism and to fulfill such pan-ethnic aspirations ( Assefa, 2022; Horst, 2020; Marzagora, 2017).

However, since the 1991, the defeat of the Derg regime by a collusion force from diverse ethnic groups changed the country’s political space from pan-Ethiopian nationalism to pan-ethnic nationalism. The aspiration of one nation and one language of the imperial and Derg regime is replaced by the EPRDF regime that provides recognition (at least in principle) for the multinational identity, which is completely different from previous political regimes/systems ( Abbink, 2011). Most research concluded that ethnic identity was used as an instrument to perpetuate the political and economic benefits of political groups and elites of the EPRDF. Moreover, as in previous regimes, the EPRDF failed to create genuine and true multinational federal states. The persistence of multiple national identities and the repression of these identities by the imperial regimes were used as political opportunism by the EPRDF government to exercise divide and rule policy since its inception ( Assefa, 2022; Monenus, 2017; Adamu, 2013; McCracken, 2004).

The articulation of multinational politics (commonly used as ethnic politics in Ethiopia) during the EPRDF regime, like its predecessors, also failed to create a truly democratic federal state in Ethiopia. It failed to balance the competing identities in Ethiopia and remains fragile in nature ( Fiseha, 2007, 2012, 2018, 2019, 2022 and 2023). However, identity has been utilized as a means of perpetuating differences without creating a strong foundation for equality and democratic-based citizenship. While previous regimes openly repressed nationality questions, the EPRDF appropriated pseudo-identity-based politics to perpetuate differences and use it as a means of divide and rule. From an identity point of view, the EPRDF’s identity politics neither resolved the national oppression thesis nor created genuine citizenship ( Assefa, 2022; Marzagora, 2017; Monenus, 2017; Adamu, 2013; McCracken, 2004). Hence, the pseudo multinational federalism of the EPRDF has been sandwiched between the proponents of pan-ethnic nationalism and pan-Ethiopian nationalism, which later made the regime unstable like the previous regimes.

Therefore, both the pre-1991 and post-1991 political history of Ethiopia signify that neither of the two extremes could be the solution for nationality questions and to create genuine and democratic citizenship. Despite the existence of constitutional provisions and policy frameworks to balance Unity (citizenship or civic nationalism) and diversity (ethnic nationalism) in Ethiopia, as provided in the FRDE constitution ( FDRE, 1995), the government of the EPRDF failed to sustain the balance because of structural and practical gaps in implementing the constitution ( Van der Beken, 2009). In this regard, Abbink (2011, p. 611) stated that despite the progress of the 20-year experiment, ethnic federalism in Ethiopia “neither compensate for the socio-political problems nor guarantee stability. At present, there still are constraints and dilemmas in the field of ethnicity and citizenship.” Moreover, Abbink (2011, 2012), Fiseha (2012, 2019, and 2023), and Belay (2013) testified to the failure of the EPRDF to genuinely implement federalism.

Moreover, the state and administrative structure was pseudo-federalist, as the central government dominated power and authority throughout the country. Agegnehu and Dibu (2016, p. 4843) concluded that “the development and consolidation of centralized dominant party rule which is paradox of genuine federalism, manipulates ethnic group in search of enlarging its power through applying a divide and rule approach.” As Keller and Omwami (2007) stated, the EPRDF government was not a true federalist and operated in a unitary state mentality. This means that multinational federalism in Ethiopia, during the EPRDF, was used as mask/sheepskin to mobilize community and consolidate power ( Assefa, 2022; Monenus, 2017; Adamu, 2013; McCracken, 2004).

Given its misappropriation and weakness in addressing the identity problems of the country, the post-1991 politics of Ethiopia indicates a complete shift from pan-Ethiopian nationalism to pan-ethnic nationalism. Viewed from the dialectical method, pan-ethnic nationalism became a thesis and pan-Ethiopian nationalism became an anti-thesis in the politics of the country. However, as in the previous regimes, this dialectic failed to create a strong and required synthesis. This means that, like the politics of its predecessors, the politics of the EPRDF regime failed to create genuine, democratic, and equality-based citizenship and failed to resolve historical problems through restorative justice. Such dialectical rotation of identity-based contradictions and opposing state formation approaches can be presented as shown in Figure 1. The figure represents the dialectical nature of Ethiopia’s identity politics in the post-1991 period.

## 4. Unending Dialectics in Ethiopian Politics

From the above analysis, it can be concluded that the processes of state formation, identity questions, self-determination, and ethnic conflict in Ethiopia are inevitable. The current political situation indicates the unending dialectics of Ethiopian politics. The existing politics of ethnicity, identity, and citizenship indicate the unending process of political friction with the rotation of thesis and anti-thesis, which left the state-building projection of the country’s unstable and weak dialectical process. This was the result of the political miscalculation of the ruling regimes since the inception of modern Ethiopia which led to lack of mutual trust between government and ethnic identity groups in one hand and among ethnic identity groups in the other. That is, the wrong policy and political response of successive regimes to the nationality questions by successive governments resulted in a sustained and vicious circle of conflict ( Assefa, 2022; Jima, 2021). This makes the politics of the country a vicious circle of contradiction and conflict since its inception as a nation-state ( Jima, 2021; Zerai, 2017).

**Figure 2.**
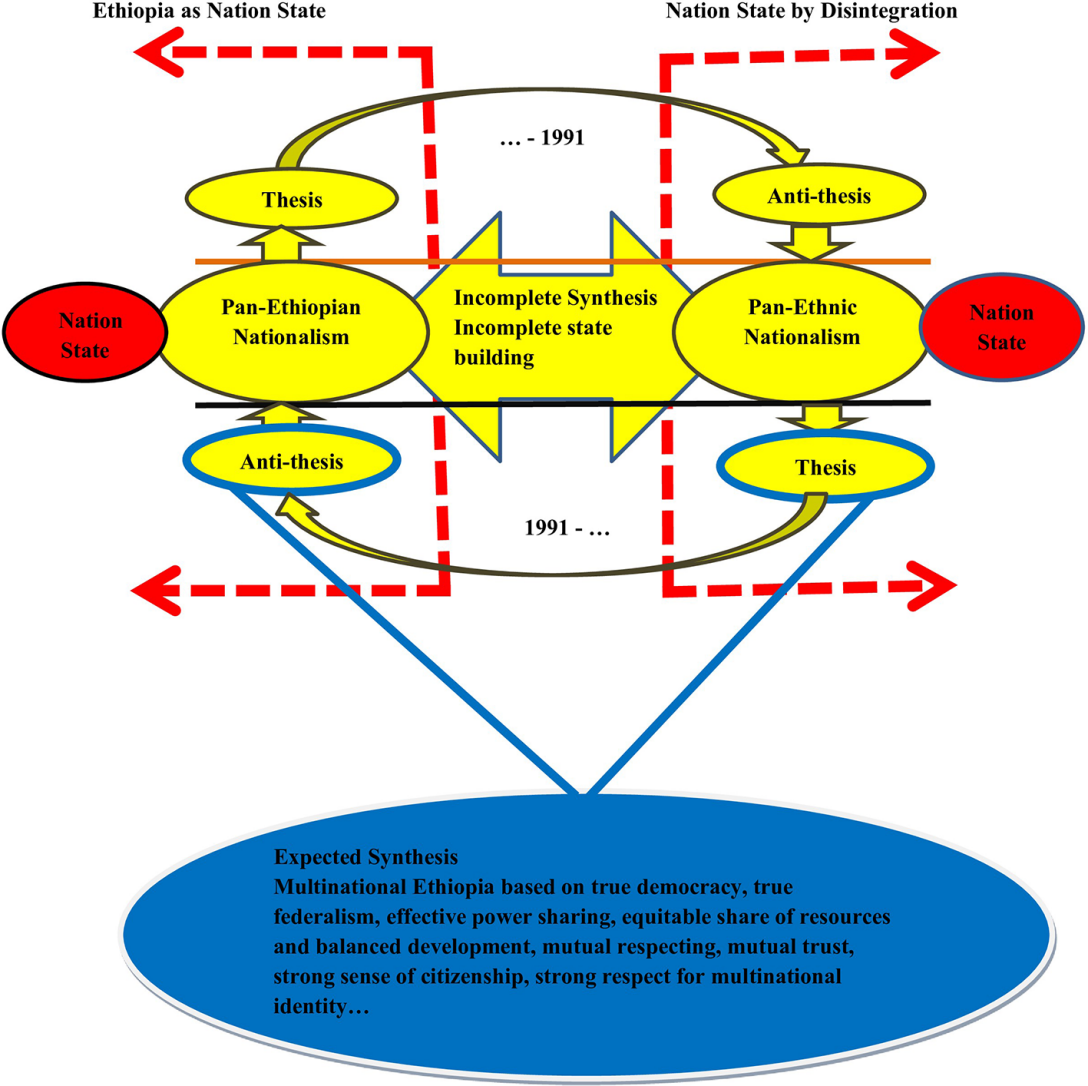
The cyclical Rotation of Thesis and Anti-thesis in Ethiopian Political History. Note: Developed by the author based on literature (2024) Keys to the Model a. Expected Synthesis: Aspiration of so many Ethiopians as general and politically active proponents of ethnicity-based identity. It is a required Ethiopia that was built based on consensus democracy, which was aspired by the majority of political elites (both pan-Ethiopians and pan-ethnic nationalists), and the community, which was inspired by security and peaceful coexistence. b. Incomplete Synthesis: This indicates incomplete state building attempted by successive regimes since the inception of the modern territory of the country as long as the contestation between the two nationalisms led to violent vicious cycle of conflict. c. Nation State Through Homogenization: Top dawn approach to nation building and state formation based on one nation, one language, and mono-national identity. The model was attempted from Minilik II until the 1991. Such an attempt is termed as aggressive nationalism, which was attempted in European countries, such as France, Germany, and Italy. d. Nation State Through Disintegration: A reciprocal of “c” which aimed at forming single nation state through secession and breaking dawn the current Ethiopia into numbers of completely autonomous and sovereign states. e. Thesis/Anti-thesis: Dialectical triads that change with changes in the political history of the country. It was the only change in Ethiopian politics regarding identity. f. The Broken Red Arrow: Indicates the criticality of identity-based contestation (pan-Ethiopian and pan-ethnic nationalism). Currently, Ethiopia is at a critical stage, as the contradiction is followed by armed conflict. The arrow to the left indicates the extreme politics of Pan-Ethiopianists (nation-building through homogenization), while the arrow to the right indicates the politics of identity based on distinct national identity and the quest for independent political entities (formation of a nation state through disintegration and secession). g. The part of the model above the horizontal black line indicates the dialectical contradiction of the country’s pre-1991 politics. h. The part of the model below the horizontal orange line indicates the dialectical contradiction of the country’s post-1991 politics.

Figure 2 indicates the cyclical rotation of Ethiopian political history based on the dialectical model. The model combined the dialectical contradiction (contested political notion) of the imperial regime with that of the political nature of the country since the 1991 with specific reference to identity politics. This indicates how the pre-1991 political history (as indicated in Assefa, 2022, p. 112) of the country contradicted its post-1991. In the model, an attempt was made to indicate how current political conditions are linked to the country’s political history. The model indicates that the Ethiopian history of identity politics and contestation failed to create a strong and new synthesis that accommodates thesis and anti-thesis ( Assefa, 2022). The initial promises of the EPRDF were to create a multinational democratic state and ensure unity in diversity; the TPLF stretched its political tentacles to control and monopolize power, resulting in political exclusion, corruption, confiscation of national resources, utilization of identity for political and economic gain, and other multi-dimensional exclusions ( Jima, 2021; McCracken, 2004).

Important questions that need to be addressed at this point are the direction to which Ethiopia is moving: disintegration, nation building through homogenization, or building a Multinational state based on genuine democracy? The current political situation indicates a strong contradiction between Pan-Ethiopianism and Pan-ethnic nationalism. Compared to the historical process of the country, the current conflict and political discourses are not in position to create the expected synthesis, as shown in Figure 2. This chaos and political crisis lead the future fate of country to be indeterminate at the current time. The country is at the bottom of multiple crises and chaos, with no clear political roadmap and vision regarding how to resolve the current political contestation. There is a conflicting vision of the country among competing political actors ( France 24, 2021).

The more critical challenge at the current time is the change in the trajectory of pan-Ethiopian nationalism into ethno-religious nationalism by extreme Ethiopianists. Moreover, the current issue of religious institutions poses additional fuel to identity-based contradictions in the country. The aim of the discourse of ethno-religious nationalism is the restoration of the greatness and dominance of specific ethno-religious groups, the so-called great tradition of imperial regimes. They argued that the greatness and dominance of such ethno-religious was lost with the demise of the imperial regime in 1974 (see Amhara Media Corporation, 2019). The marriage between the religious elite and political elites at the current time changed the nature of political discourse in the country, which might be a critical juncture for religious contradiction, conflict, and additional political ferment. Such a changing trajectory might exacerbate the historical and contemporary political crisis, adding new fuel to the existing problem of identity politics.

## 5. Implications

The history of Ethiopia indicates that the majority of political contestations of the country constitute identity (ethnicity) dimensions. Conflict of resource ownership, history of the country itself, political polarization, questions of political participation and representation, government responses to political questions, state formation and state-building projects, and other government decisions are seen from identity (ethnicity) perspectives. The country is still sandwiched between extreme identity-based political discourses, which are a source of major political problems in the country. The country is sandwiched between two extreme nationalisms (including both pan-Ethiopian nationalism and pan-ethnic nationalism).

This makes the politics of a country prone to a vicious circle of contradiction and conflict, which hampers desirable state formation and building. Hence, the country currently has three political options: the formation of a nation-state through homogenization, the formation of a nation-state through disintegration/forceful dissolution, and the formation of a democratic multinational state. Now, some of the political discourses of so-called pan-Ethiopian nationalism have changed to ethno-religious nationalism, in which some elites quest for ethno-religious dominance in the politics of the country. Numerous pseudo-pan-Ethiopian nationalists call upon religious, political, and ethnic dominance in the country. Still, there are numerous political elites in the middle of the road, which gave ample opportunities to create a consensus based multinational state.

This further implies that Ethiopian history and the current political landscape indicate that the prescription of one side of nationalism at the expense of the other will never resolve the political crisis and a vicious circle of contradiction in the process of building strong and inclusive state institutions. Ethiopians’ historical and ongoing crises are rooted in the history and process of creating and remaking Ethiopian polity. Professor Merera calls this the transition from crisis to crisis. In Assefa (2022), this crisis is called a vicious circle of conflict, and Jima (2021) in his article, Vicious circle of Ethiopian politics: Prospects and challenges of current political reform, he calls this a vicious circle of Ethiopian politics. McCracken (2004) calls Ethiopian history crisis history. This indicates that Ethiopia has inherited political crises throughout its history, which is characterized by an unending dialectical contradiction between pan-Ethiopian nationalism and pan-ethnic nationalism.

## 6. Recommendations

Therefore, a balanced view of identity based political discourses, claims and problems in the country is a primary condition to be nurtured in the political discourse of the country, if one needs to reduce the actual and potential ethnic conflict in Ethiopia. In particular, considering the current political discourse of the country, it is recommended that the existence, recognition, and institutionalization of both identities is a prerequisite to reverse the conflicting history of the country and ensure peaceful coexistence for the current and future generations.

Scholars of identity politics recommend that there should be a distinction between nationality and citizenship. Given the subtlety of the distinction between nationality and citizenship, such a separation has important implications in resolving identity problems that emanate from a faulty definition and misinterpretation of identity in Ethiopia. As a multinational state, there should be coexistence of civic identity (citizenship) and national identity (Nationality) in Ethiopia. Hence, there should be a genuine definition of identity that could reverse the antagonistic relationship between the pan-Ethiopian (faulty consider Ethiopia as a nation) and pan-ethnic proponents. Hence, mutual recognition of both citizenship and nationalism is recommended. Regarding this Piattoeva (2016: np) conclude that “Citizenship must now reconcile the initial pursuit of equality and universality with the recognition of difference. This question is essentially about whether the two concepts can be perceived in a multilayered fashion, as opposed to viewing them through a zero-sum game logic.”

What Ethiopia needs is, therefore, the creation and strengthening of democratic rules, institutions, the environment, and policies in which one’s own identity can be positively and equally treated without infringing on others. There should be mechanisms that prevent the forceful attempt to impose one’s own identity or the identity that one likes over another, because no one is the maker of identity for another. This is because democracy needs free human choice with which individuals make their own identity choice and enjoy identity rights without infringing on others. A thoughtful and wise political discourse is needed to manage identity tension among elites in the country. In particular, considering the current political situation of the country, one should not carelessly repress claims for representation, self-determination, and self-administration, and quests for identity recognition raised based on multinational identity.

Both historical and current political situations in Ethiopia indicate that Ethiopia is a multicultural state in which more than 80 (87 nations according to research conducted by the Ethiopian Nationality Study Institute in 1987) nations persist. Not only persists but also indicates the impossibility of assimilating/repressing these multinational identities along the narrow definition of Ethiopian nationalism. Ethiopian nationalism ( የኢትዮጵያ ብሄርተኝነት ) is a paradoxically created identity by the political elites of the so-called dominant culture/group of the imperial regimes. The historical and current political problems of the country have emanated from an attempt to impose narrowly defined nationalism. Hence, the application of a more complete dialectical approach in dealing with the antagonism between the proponents of Ethiopian nationalism and multicultural nationalism is also important for harmonious coexistence and successful state-building. As shown in Figure 2, it is recommended that the expected synthesis, which is detached from the unending dialectical contradiction between Pan-Ethiopianism and Pan-ethnic nationalism, should be Greater Ethiopia, in which both citizenship and nationalism are respected based on mutual understanding and belongingness.

To this end, well-known scholars of federalism, Fiseha (2023), recommend the application of both integrative and accommodative federalism to balance the two extreme quests, integration for pan-Ethiopian nationalism, and accommodation for pan-ethnic nationalism in the country. Assefa and Wami (2023) recommend the realization of genuine and real multicultural democratic federalism rather than mobilizing citizens from an instrumental point of view.

Moreover, the complimentary method of the Switzerland federation should also be implemented to protect the rights of intra-regional minorities, which Ethiopian federalism practically failed to protect. Switzerland could be the best example where cantonal-level minorities are protected without contravene cantonal autonomy and self-rule. According to Belser (2018), the European Framework Convention and the new framework for protecting national minorities have been taken seriously by Switzerland, without curtailing cantonal and municipal authority. The idea of national minorities has not been utilized to replace established methods of accommodating minorities but rather to enhance them. Therefore, Ethiopian need to install some legal and institutional design to protect the right of intra-regional minorities.

## Data Availability

The data used in this research were obtained from secondary sources. Raw data was not collected for and used in this particular article.
