# Unending dialectical politics of identity in Ethiopia

**DOI:** 10.12688/f1000research.160666.2

**Published:** 2025-12-27

**Authors:** Tefera Assefa

**Affiliations:** 1 Center for Federalism and Governance Studies, Addis Ababa University College of Social Sciences, Art and Humanities, Addis Ababa, 1176, Ethiopia; 2 Public Administration and Development Management, Ambo University Woliso Campus, Woliso, Oromia, Ethiopia

**Keywords:** Ethiopia, Pan-ethnic nationalism, Pan-Ethiopian nationalism, Identity politics, unending dialectical

## Abstract

**Background:**

Identity politics is one of political terminology which is subjected to continuous and increasingly contested conceptualization and use. Moreover, identity exists in all social level starting for individual to international level, which makes identity a multi-layer concept. There has been a great deal of identity-based terminological distortion and misconception in Ethiopian political discourses and practice since its formation in the modern form. One of the primary challenges in Ethiopian present political discourse is misconception, complexity, and contradiction between ethnic identity (የብሄር ማንነት) and civic identity or citizenship (የዜግነት ማንነት), which must be addressed in Ethiopian political discourse.

**Methods:**

In this paper, critical post-modernism research philosophy and Dialectical Method is used to analyze the rotation of contradiction between Pan-Ethiopianism and Pan-ethnic nationalism. The narrative analysis is used to make a critical investigation of the vicious cycle unending dialectics of identity politics in Ethiopia.

**Discussion:**

The current political situation is an indication of unending dialectics of Ethiopian politics. The existing politics of ethnic identity and citizenship indicate unending dialectic of political friction with the rotation of thesis and anti-thesis which left the state building projection of country incomplete and weak dialectical process. Wrong policy and political responses of successive regime to the nationality questions (quests for power sharing and autonomous self-government) resulted in the sustained and vicious circle of conflict.

**Conclusion:**

Generally, it is argued that prescription of one side of nationalism at the expense of the other will never resolve the political crisis of the country. Therefore, it is recommended that both integrationist and accommodationist institutional design and multinational democratic federalism should be implemented effectively to cope with the salience of identity politics in the country.

## 1. Introduction

Identity politics ^1^ is political terminology that is subjected to continuous and increasingly contested conceptualization and use, mostly in developing countries. Moreover, identity exists at all social levels, starting at the individual to international level, which makes identity a multi-layer concept in which multiple identities coexist at each level and the salience of any particular identity ebbs and flows depending upon the circumstances ( Phillips, 2016; Muir & Wetherell, 2010). In modern political theory, identity politics is an integral part of both the conflict and peace-building processes ( Orjuela, 2008; Heywood, 2019). More importantly, identity politics are also used as an instrument of nation or state building most of the time used by the dominant group ( Leach, Brown & Worden, 2008). However, most of the time, the role of identity and its political manipulation by dominant group is not considered as identity politics, and it is mistakenly linked to and equated with the politics of minority groups that look for liberation from social, political, and economic repression ( Holberg Prize, 2021).

Along with strong identity-based contestations, there have been many identity-based terminological distortions and misconceptions in Ethiopian political discourse and practice since its formation in the modern form. In Ethiopian political discourse, there is uncertainty over ethnic identity, racism, and ethnic prejudice. Any definition of racism cannot encompass Ethiopia’s past and present quests for ethnic identity (identity politics). According to various definitions of racism, the quest for ethnic identity recognition and self-determination in Ethiopia in the past and now cannot simply be equated with racism and ethnic prejudice. However, the extremist political elite (the pseudo pan-Ethiopian nationalists for instance) consider the quest for alternative national identities as racism ( ዘረኝነት ). Furthermore, in Ethiopian political discourse, there are persistent tensions between nationality and citizenship. Treating these two phrases as synonyms is a risky political calculation, especially in multinational and multicultural countries.

According to theoretical and empirical data, the link between ethnicity (nation/nationalism) and citizenship is complicated and complex ( Sant, Davies & Santisteban, 2016; Hristova & Cekik, 2013). Nationalism and citizenship are used interchangeably. However, in both theory and practice, these two concepts are distinct. Several scholars have discussed the relationship between nationalism and citizenship. Some academics correlate nationalism with citizenship, whereas others seek to establish a relationship between the two categories. Others draw a clear contrast between the two terms, while others associate nations with the modern nation-state, in which nation and state coexist/coincided ( Piattoeva, 2016; Kaufman & Williams, 2017; Indian Today, 2018). “A lot of the confusion stems from the fact that ‘citizenship’ is usually confounded with ‘nationality/nationalism’, and sometimes the two, conceptually different, terms, are regarded as identical and used interchangeably” ( Meehan 1997 cited in Tsaliki, 2007, p. 162) and nation-building has been used by Jacobin unitarists to build nation-state from multinational community ( McGarry and O’Leary, 2009 and 2005). However, the two terms are significantly different from each other. Particularly in multinational or multicultural countries, it is a dangerous political calculation to treat the two terms as synonyms. In particular, with the advent of the multinational modern constitutional state, the difference between the two terms became clear. To quote the words of Tsaliki (2007, p. 162), “at the dawn of the modern constitutional state, the two terms differed greatly.” Moreover, the old model of the nation-state project failed with the emergence of a quest for alternative identity, which led to the development of multiculturalism and multinational federalism ( Kymlicka, 2007). Scholars such as Parekh (2008) and Laclau (1992) conceptualize the complementarity of identity politics based on dialectical ^2^ nature of universal and particular identity politics. Parekh for instance, considers that both common human identity and particular identities as crucial in the life of the community. His argument is that both communalism and Particularism should be seen as an integral and complementary to one another. Parekh also argued the impossibility of disentangling of the universal human identity from that of particular identities, because human solidarity is built on the different political, cultural and other communalities which provide moral energy. Hence, he considers the particularity and universality as complimentary, not conflictual to one another and calls redefinition for the harmonious relationship between the two.

Generally, the universal is manifested by its particularity while particularity is art of universal ( Laclau, 1992). When addressing the dilemma of Particularism and universalism, Laclau (1992, p. 85) also stated that “universality can only be a particularity that defines itself in terms of a limitless exclusion; while the particular itself becomes part of the universal, and the dividing line is blurred.” Taking the universalizing particular as evidenced in the European universalization of their particular identity, he conclude that the universal is the attempt by the particularist to universalize their particular identity. Argument here is that the universal identity by itself is evolved from particular identity. Based on extensive theorization of universalism and Particularism, Laclau argued that the conclusion seems to be that universality is incommensurable with any particularity yet cannot exist apart from the particular. Laclau continued that the relation between Particularism and universalism is paradoxical in which various groups vie to provide a transient function of universal representation for their specific goals. What is problematic is that the inability of society to finally define itself as society—which is equivalent to failing to recognize difference as difference—makes the gap between the universal and the particular unbridgeable which make democratic interaction impossible (ibid) which led to clash of nationalism and violent conflict in some instances. Such problem is well conceived and evidenced in the political history of Ethiopia and the current political crisis is the manifestation of lack of willingness to recognize difference as difference.

In Ethiopia, nationalism and citizenship cannot be used interchangeably. This is due to the fact that Ethiopia could not be established or developed as a nation (nation-state). In Ethiopian history, there has been major elite-based tension between nationalism and citizenship, which stems from a poor definition and understanding of the two categories. Ethiopian political conflict of identity is defined by a vicious loop of contradiction between thesis and anti-thesis ( Assefa, 2022) which failed to recognize the complementarity of citizenship and nationality (ethnonational identity politics). The political history of the country failed to consider the complementarity of both ethnonational identity and civic identity as conceptualized by Parekh and Laclau. This further leads to failure to recognize difference as difference, which make identity based contestations unbridgeable and cause violent conflict.

As a result, one of the primary political challenges in Ethiopian present political discourse and history is the misunderstanding, complexity, and contradiction between ethnonational identity ( የብሄር ማንነት ) the so assumed particular identity politics and civic identity or citizenship ( የዜግነት ማንነት ) the so assumed universal identity politics, which must be addressed in Ethiopian political discourse. On the one hand, the slogan of one nation, one language, one religion, and one identity remain visible, while on the other hand, there is a rising proclivity for ethnic-based identification. Lyons (2019, p. 3) clearly put that “Ethnic identity and Ethiopian identity have an extensive scholarship and are key parts of contemporary Ethiopian politics.” Such confusion should be critically dealt with to create an all-inclusive and mutually agreed upon democratic citizenship in which both identities are truly recognized rather than building one at the expense of others. In Ethiopia, the two version of identity politics is considered as incompatible and any government efforts in the process of state making/building resulted in continuous political crisis and conflicts. This article attempts to answer how the contestations based on identity politics in state making process embedded in the post-1991 politics which further resulted in unending dialectical contradiction between pan-Ethiopian nationalism and pan-ethnic nationalism? Hence, this article aims to critically analyze the unending dialectical contradiction between pan-Ethiopian and pan-ethnic nationalism which led to violent conflict.

## 2. A brief political history of Ethiopia

There are scholarly debates and contestations regarding the root of identity-based politics and conflict in Ethiopia. Numerous scholars in Ethiopian politics and history argued that imperial regimes remained antecedents for immediate and prospective identity-based struggle for power ( Assefa, 2022; Berhe and Gebresilassie, 2021; Horst, 2020; Gudina, 2011). Historical narration and legacy of the imperial regime constituted to be one of antecedents of ethnic-based conflict in Ethiopia since its inception as a modern polity ( Jima, 2021; Gudina, 2011; Jalata, 2010; Gebru, 2009). However, some scholars such as Ishiyama (2023) argued that identity-based politics and conflicts are rooted in the post-1991 politics of the country, with some extension to the 1960s student movement and the demise of the imperial regime in 1974 ( Halabo, 2019). However, looking at post-1991 politics of the country without historical accounts leads one to a partial conceptualization of identity politics and its impact in the process of nation building in the country. Therefore, one need to make a comprehensive analysis of the implications of ethnocratic politics since the imperial regime of Ethiopia and how identity politics is narrated in the post-1991 political discourses of the country. It is stated that the imperial regimes left the country with far-reaching political, social, and economic difficulties that have ramifications for the present and future/potential identity-based conflicts ( Assefa, 2022; Gudina, 2011) and actually manifested by multiple political crises and violent conflict that ravaging the country since 2018.

Given the reality, some scholarly writing on identity politics, with special reference on multi-national identity politics (commonly referred to ethnic identity politics in Ethiopia), in Ethiopia concludes that the post-1991 identity politics is the sole causes for the contemporary identity-based conflict in the county (see for instance Ishiyama, 2023; Atnafu, 2018; Nahoo TV, 2018). Numerous writers and political elites limited their analysis and critics on the quest for alternative identity and consider identity politics as a curse for peaceful coexistence. Hence, such literature excludes the pre-1991 politics of the country from the conception and inquiry of identity politics which still remain as a source of critical political problems throughout the country.

The pre-1991 politics of the country is in sharp contradiction with what Fessha (2016, p. 151) said, citing Conti Rossini’s famous words ‘Un museo di popoli’ which indicates multiethnic, multilinguistic and multifaith character of Ethiopia. It is also contradicting with what Moreno and Colino (2010) conclude that diversity is not a threat for the survival and prosperity of federal countries and recommends that recognition and accommodation of diverse identity is a base for legitimacy, national unity and social cohesion. Moreover, the imperial projection of nation-building had also been contradicting with the 2001 Universal Declaration of Cultural Diversity proclaimed by UNESCO ( Kymlicka, 2007; UNESCO, 2001). This is why most European countries now resorted to recognize quests for autonomous self-government by numerous territorially defined identity groups. Catalonian in Spain, Scotland, Irish and Wales in UK, and advancement of Belgium to federalism are the example where the salience of identity and its attendant politics led to decentralized and federal forms of government. Considering the impossibility of the imposition of the so-called dominant identity on the rest of the population and the failure of the traditional political institution to respond to the increasing quests for recognition and autonomous self-rule, western countries are now resorting to the alternative institutional design. For instance, Erk (2008), based on empirical study on Austria, Belgium, Canada, Germany and Switzerland, states that political institutions have gradually changed to reach a better fit with the ethno-linguistic social structure.

In most of its history, Ethiopia has been suffering from continuous political crisis and civil war. The successive regimes have never been able to overcome these issues until now. They rather contributed their parts to the historical problems of the country which is exacerbated by the use of force (naked power) in the struggle for attaining and remaining in power ( Assefa and Wami, 2023). Misappropriation and mis-conceptualization of identity politics as manifested by problems of minority rule, minoritization of major ethnic groups, negative political discourses, lack of equitable political repressions, lack of development, political opportunism and unbalanced resource distribution. These are some of the problems that arose from the country’s imperial ethnocratic regimes and aggravated throughout the history of the country which still salient in the country’s politics ( Fiseha, 2005, 2024). Governments have failed to create genuine political environment/system that effectively deals with quests for identity recognition, political representation and self-determination by numerous ethnonational groups. In Ethiopia, long-term violence is frequently the outcome of political miscalculations ( Assefa, 2022; Berhe and Gebresilassie, 2021; Fiseha, 2005) and misappropriation of identity and its attendant politics in the process of nation-building. The problem has been the application of ethnocratic politics in the allocation of power and resources, regime in regime out, since the inception of the modern Ethiopia.

## 3. Theoretical framework

As identity politics is characterized by the dialectical contestations in which at least two contending views are contradicting one another for the sake of achieving their respective aims. Identity in its nature need to be contrasted with its opposition (white and black, and women and men for instance) to be a meaningful. But at the same time the oppositions are interdependent and occur within the same/common social system. This is considered as a strategy to protect once identity from repression by the dominant groups with vested interest in expressing communalities ( Parekh, 2008). Parekh also argued that the fundamental opposition is not merely between the identities, but also between “the identities in one hand and the wider social and cultural structure on the other hand, which both have a common emancipatory interest in changing” (p. 40). This intern lent the dialectical nature of identity politics as dialectical is concerned with contradiction of phenomenon in one and unified realms. This is why Parekh argued that politics of identity need to go beyond confrontation and polarization. He asserted that politics of identity need to find a way to forge a wider cultural and political coordination.

In Ethiopia identity politics is used a means of allocation of political power and design of state structure throughout its history and the process of its formation and reformation ( Vaughan, 2003) which resulted in the identity based contradictory discourses that dominate the politics of the country. The two major contending views are based on the question of nationality. This has been one of the major problems in Ethiopia which emanated from misappropriation and miss-conceptualization of identity politics while at the same time it is used as means to claim for power sharing and designing state structure (one contradiction in identity politics of the country). There have been conflicts between the proponents of mono-nationalism and multi-nationalism which are rooted in contradiction between the positivist and postmodernist aspirants of identity (the second contradiction).

The history of identity politics in Ethiopia is characterized by unending dialectical rotation between thesis and antithesis ( Assefa, 2022) full of not only the crises but also a vicious circle of crises ( Assefa, 2022; Jima, 2021). Since the inception of the multi-national character of the country, it has been experiencing continuous and sustained political instability. The current political situation is an indication of the unending dialectics of Ethiopian politics. The existing politics of ethnicity, identity, and citizenship indicate an unending process of political friction with the rotation of thesis and anti-thesis which left the state-building projection of the country incomplete dialectical process. This is the result of political miscalculation of the ruling regimes since the inception of modern Ethiopia. That is the wrong policy and political responses of the successive regime to the nationality questions by a successive government which resulted in a sustained and vicious circle of conflict ( Assefa, 2022; Jima, 2021). These make the politics of the country a vicious circle of contradiction and conflict since the inception of the country ( Jima, 2021; Zerai, 2017).

Such failure is explained as “a grand failure” by the prominent political scholar of Ethiopian politics, Gudina (2003). Gudina identified five grand failures of the successive regime in Ethiopia. These five grand failures have been rooted in the history of making and remaking of Ethiopia by successive regimes. The primary reason for grand failures is the faulty empire-building calculation which was designed based on identity categorization of the imperial regimes and later a faulty response to the past identity categorizing. Therefore, to critically asses the vicious cycle of contestations and political crises, the Hegelian dialectical triad was used as a conceptual framework. As dialectic is the philosophy of totality, the researcher will never limit its perspective to either aspect of a contradiction. Fuchs and Sandoval (2008, p. 114) for instance put that “dialectics is a philosophy of totality means that society is analyzed on a macro-scale to grasp its problems and that reasons for the necessity of positive transformations are to be given.”

As ethnic identity politics by itself is a dialectical struggle between the dominant group and the subordinate group, as stipulated in Leach, Brown, and Worden (2008), the application of the dialectical inquiry (DI) is most appropriate for this research. Berniker & McNabb (2006, p. 646) argued that “the logical structure imposed by DI requires the researcher to identify competing models and explore them in depth.” As noted by Assefa (2022), the historical narrative of Ethiopia is marked by a dialectical process wherein multiple opposing perspectives have consistently clashed throughout its development and formation. He also recommends the application of dialectical approach to investigate two facets of the quest for identities in Ethiopia. Therefore, he contends that a dialectical approach provide a new insight in the conceptualization of the contradiction of the quest for identities and reach viable conclusions on the current real and potential political volatility. Scholars like Canivez (2019) use dialectic to grasp the real in all of its diverse and contradictory interpretations. He sees dialectic as a method that comprehends the real as a whole and explains how such understanding is even possible. However, the adjusted logic of the dialectical method is used in this article. Unlike the dialectical logic of Hegel, Marx and Engel, which emphasizes on linear historical progression/development, which emphasizes on the absorption of contradicting idea into a wider idea in which they live without contradiction ( McTaggart, 1922), this article uses the cyclical rotation of the dialectical triad to indicate the vicious circle of identity-based contractions that characterize Ethiopian unending/vicious cycle of identity based political discourses and conflict. Based on this, the article design and use a new model that shows dialectical rotation/a vicious cycle of identity politics of the country. This model could also be used in another settings and fields (as it is or modified) to conceptualizes a vicious cycle of contradictions.

## 4. Methods

In this article, secondary sources of data, such as books, articles, working papers, reports, videos, audios of speeches and interviews held by different media, were used to indicate how the post-1991 narration is rooted in the imperial projection of nation-building and the sustainable contradictions and contested nationalism between pan-Ethiopian and pan-ethnic nationalism. The number of secondary material used is selected judgmentally based on the relevance and sufficiency method. Narrative and qualitative trend analysis is used to indicate how pan-Ethiopian nationalism has shifted to ethno-religious nationalism discourses held by politico-religious elites and the response to the narrations by the proponents of multi-cultural nationalism and the exacerbation of the quest for autonomous self-rule. Therefore, Dialectical critical post-modernism of research philosophy and qualitative research approach is used to analyze the rotation of the contradiction between Pan-Ethiopian and Pan-ethnic nationalism. Moreover, an attempt has been made to develop a dialectical model that shows the political discourse of the country since the 1991. Moreover, the pre-1991 dialectical model of Assefa (2022) and post-1991 dialectical models are combined to develop a comprehensive dialectical model that indicates the cyclical rotation of thesis and anti-thesis and the unending dialectic of Ethiopian identity politics, which results in a vicious circle of ethnic conflict in the country ( Assefa, 2022; Jima, 2021). The year 1991 was taken as a critical juncture that resulted in a complete shift of the post-1991 nationality/identity discourse and brought identity politics into the formal legal, institutional and policies in the political history of the country.

## 5. The post-1991 political discourses in Ethiopia

The political watchwords at the current time (post-2018) revolved around nation building, which emanated from the faulty definition of state and nation. Throughout the historical and current politics of Ethiopia, it is common to observe the misconceptions of nation-building and state-building. Nation-building discourse could not be a solution for the sequences of past political mistakes, current political problems, and for future state-building and peaceful coexistence in multinational states like Ethiopia. Ethiopia is a multinational state that has no single national identity but identities that should/need to be recognized, which have never been genuinely recognized throughout its history and in contemporary politics of the country. Here, the political headache of the country is not only a contradiction between the quest for such multinational identities and a single national identity, but also the ways in which the contestations is treated by successive governments ( Assefa, 2022; Gudina, 2011; Jalata, 2010 and 2020; Jima, 2021).

The rejection of the desire for a multinational identity (which is based on the particularistic identity) by the ethno-religious elite and pseudo-Pan-Ethiopian nationalists (which use common humanity as an argument to challenges the claim for multi-national identities) exacerbated ethnic hatred and strife. Along with this, the country’s post-1991 politics failed to create a genuine multinational federalism and to address nationality issues in meaningful ways. As a result, there is a plethora of assertions that ethnicity is controlled by the political elite and is utilized as a tool of divide and rule ( Gedamu, 2021). It is thought that multinational federalism was used as a pretext by the TPLF elites to influence and mobilize the community for political and economic benefits by the EPRDF/TPLF ( McCracken, 2004). For example, one can easily imagine how the EPRDF government unconstitutionally responded to numerous ethnic groups’ constitutional quest for self-determination in the SNNPs region (the case of the Sidama People, Silte and Gerageh, Wolayita, etc.) and other parts of the country, implying a lack of genuine implementation of true federalism elsewhere in the country, resulting in violent ethnic conflict, numerous deaths, dislocation, and devastation ( Dessalegn & Afesha, 2019; Halabo, 2019 & Belay, 2013).

Numerous ethnonational groups quests for autonomous self-government within the federal dispensation. The reason behind, as Girma (2020) contended, is that for the past 25 years, the federal government has failed to develop a solid institution and system that firmly answers to the country’s many ethnic identity needs. The demand for a separate identity and the right to self-determination has grown throughout time, originating from various parts of the country. For this reason, Kleppe (2022) argues that the dispute in Ethiopia is about the status and acknowledgement of nations and ethnic identities. In Ethiopia, despite the whole country experiencing some political crisis that emanates from the deliberate use of identity for political purposes, the problem is more critical in some regions since the inception of the Ethiopian federation. These regions include Oromia National Regional State, Amhara National Regional State, the former Southern Nation, Nationalities and Peoples Regional State (SNNPRS), and Tigray National Regional State (particularly post-2018). These four regional states are well conceived as a hub of identity-based claims or quests, at least for power sharing and genuine implementation of the ethnonational federalism, and at most the redesign of the federal structure and secession. In addition to strong territorial cohesiveness and concentration, ethnic mobilization and counter-mobilization are high in these regions. Political events in these regions have national and international ramifications. Moreover, there has been contestations between various political actors regarding the federal design in Ethiopia ( Gebeye, 2023) which led to the political crisis and now armed conflict between ethnonational groups in one hand and between ethnic based political factions and government in the other hand.

For instance, in the former Southern Nations, Nationalities and Peoples Regional state (SNNPRS) different ethnonational group quests for recognition, autonomous self-government and regional statehood since the inception of federalism in Ethiopia ( Dessalegn and Afesha, 2019). Sidama is the best example in this regard. According to Assefa et al. (2023), the federal design amalgamated almost 50 ethnonatonal groups under one regional state that led to continuous resistance and ethnic based conflicts ( Berhe and Gebresilassie, 2021) For instance, the Sidama people, which now gained a regional statehood status, continuously resisting the central government ( Asmare Aragaw, 2024) which led to multiple conflicts which cause numerous death and destruction of resources. While the ethnonational groups in this region claims for more and more of autonomous self-government, the central/federal government denied their quests and impose their policy aspiration through the top-dawn design of federal structure in the former SNNPRS ( Assefa et al, 2023; Legide, 2019; Aalen, 2011; Boni, 2020; Halabo, 2017) which causes multiple conflict. The prominent event that signify the resistance to top-dawn federal design is the one that led to massacre of the Sidama people, known as “looqe massacre” in 2002 in which federal authorities killed a number of unarmed protesters ( Tronvol et al, 2020). The quest for recognition and autonomous self-government by ethnonational groups is also evidenced in numerous zones and woredas of the former SNNPRS and the 2018 political changes and crises open a Pandora box which later led to the restructuring of the region into four regional states after 30 years of federal experiment ( Deressa, 2024).

Another prominent political opposition and identity based claim has been evidenced in Oromia. Scholars such as Tolla and Royo (2022), argued that the post-1991multinational federalism has been an exclusive politics as it excludes a major political contenders specially the Oromo Liberation Front (OLF) in which a de facto one party control power apparatus. Since then, despite sporadically, the political resistance to the regime by Oromo elites and youth is caused by political repression and quest for an increasing Oromo participation and representation in government institutions to the least and Oromo liberation to the most. Initially, the Oromo resistance to EPRDF government is very limited in its intensity and scale. However the intensity and scale of resistance and youth struggle in Oromia changed and ignited in 2010s. In this respect Forsén and Tronvoll (2021) rightly put that; in 2014, the Oromo ethnic-based social movement known as Qeerroo (‘youth’) movement started major protests, demanding political responsibility and more Oromo participation and authority within the ruling coalition. For several years, widespread demonstrations erupted, forcing the party head and prime minister to resign in 2018 so that an Oromo leader could take office.

As Forsén and Tronvoll (2021, p. 3(19)) argued, “the Qeerroo used ethnic discourse as a mobilizing tool” in their political resistance to the existing power structure of EPRDF. This means that, according to Tolla and Royo, as well as Forsén and Tronvoll, the country’s politics after 1991 and the ongoing Oromo protests, in terms of the quest made by ethnonational identity, which uses identity discourse for their political purposes is defined as identity politics. Even when the Oromo were presumed to be represented in the government and the EPRDF alliance since 1991, Forsén and Tronvoll said, the feeling of alienation from authority remained. The arguments put out by the leaders and founders of the protest movement both inside and outside the country were thus based on the Oromo ethno-political platform. The opposition in Oromia remained after 2018, since Oromo’s political group was dissatisfied with the ruling party’s consolidation of power. According to a Landinfo (2023) study, the consolidation of power at the federal and regional levels by the governing party has resulted in widespread antagonism toward regional and federal officials in Oromia. As a result, the Oromo resistance to the current power structure represents both vertical and horizontal political opposition. Referring to the displacement of the Oromo from their land and the Addis Ababa Master Plan, Kelecha (2021) argued that the Oromo protests were a response to the grabbing of the Oromos farmer land. Tolla and Royo (2022) asserted that the 2015 protest in Oromia enjoyed huge support from the Oromo community, and were articulated under the rubric of Oromo identity, which is ignited by land grabs as one of its key causes. This also manifested by conflict between Somali and Oromo which has been ignited since the inception of federalism because of lack of flexible mechanism to deal with inter-ethnic division and utilization of resources and identity based territorial claim and counter claim, and quest for monopoly control on certain district and towns such as “Moyale, Mieso, Babile, Chinaksen, Gursum, Mayu Muluke, and Gurra Dhamole” (see for instance Wakjira, 2023, p. 6).

According to Pausewang (2009), the Oromos are the single group that is most exposed to control and repression. He continued that the federal structure introduced in 1991 as a response to resistance has not been able to soothe the trauma the Oromo suffered after a century of domination, dispossession and relegation to the status of landless serfs or tenants, and suppression of their language and culture. Despite the Oromo nationalist group being initially represented in 1991 politics, the latter political intimidation, arrest, and killing of the members of OLF led to exclusion from the 1992 election ( Trueman, 2001). All these are defined in terms of Oromo national identity (the Oromo nationalism, the Oromuma) ( Jalata, 2016).

The Oromo Support Group reports that the Oromo nationalism was taken as a great threat to the ruling party, EPRDF. Hence, there exists high-level detention, extrajudicial killing, torture, and disappearance of those who are suspected to be supporters of OLF ( Trueman, 2001, 2022). The complaint statement by the Oromo Support Group Australia (2009) identified 17 types of torture that the EPRDF applied to the individuals (perhaps their families) that were assumed to be supporters of OLF and argued by testifying from Seye Abrha’s, who was detained for six years in Kaliti prison, speech to the Ethiopian diaspora in the USA in 2008 that the prison speaks Afan Oromo and 99% of the prisoners in the prison were Oromos. The research conducted by The Advocates for Human Rights (2009) indicates that by the end of 1993, EPRDF arrested around 20,000 suspected OLF members and forced its leaders to exile with the aim of neutralizing the political influence of OLF in Ethiopia and Oromia. Moreover, Hassan (2001), cited in Jalata (2016), estimates that between 1992 and 2001, about 50,000 killings and 16,000 disappearances (euphemism for secret killings) took place in Oromia; he also notes that 90 percent of the killings were not reported. This is why Oromos are considered as impoverished and powerless, numerically a majority yet politically a minority in Ethiopia (Jalata, 2016). additionally, in 2015, as the protest to Addis Ababa Master plan exploded federal government killed over 230 Oromo and detained over 50,000 (see Human Right League of Horn of Africa, 2016).

Advocacy4oromia (2025) indicates based on the Oromo Support Group report, that the humanitarian catastrophe in Oromia continued in post-2018 by multiple actors such as government military force, fano political factions and others According to Østebø (2025) civilians in Oromia face threats from multiple actors, including government forces,insurgents, and criminal groups. Reports of kidnappings for ransom, inter-communal violence, and property destruction are common. Østebø added that human rights abuses are pervasive in Oromia, affecting civilians caught in the crossfire between government forces and insurgents. Arbitrary arrests, forced disappearances, extra judicial killings, and torture are widespread. The Oromo opposition to the federal government riches its climax in 2014 when the central government designed Addis Ababa master plan, which the Oromo elites consider it as a systematic means of confiscate land and displacing farmers from their land. The ramification of this opposition led to the change in the political landscape of the country. However, the trends and scale of opposition has been changed to violent armed conflict since 2018. Despite the political elites and faction of the Amhara accuse the current regime as the government of the OLF in particular and the Oromo in general, the reality shows numerous Oromo political elites and the political faction are against the regime. This could be manifested by numerous political events in which the former political elites of PP such as Lema Megersa, Taye Dendea and Milkessa Midhega who aspire the structural building, unification and protection of the Oromo need later turned against the party as if it is not representing the interests of the Oromo ( VOA Afaan Oromoo, 2019; OMN, 2023a and b, 2021, 2019)

Another prominent example that indicate the quest for identity based recognition and self-determination in post-1991 is the issue of Kimant. Belay (2013) demonstrates how the Kimant People’s identity was suppressed during imperial regimes in the name of Amharization and Christianization, resulting in one of the hottest political frictions that leads to violent identity-based conflict between Amhara and Kimant, Kimant and Tigray, and Amhara and Tigray, while Kimant claims their distinct identity (they claim that they are neither Amhara nor Tigre), sandwiched between the claim of Amhara’s and Tigray’s quest for dominance in the land of the Kimant. This claim indicates how the pre-1991 politics of the country is embedded in the post-1991 politics of the country. According to Belay (2013), Kemant’s current claim is deeply based in the injustice committed throughout the time of modern Ethiopian state-formation, which began in the mid-nineteenth century and never resolved until now.

In addition to the aforementioned issues, there is a propensity to preserve the dominant culture, which is based on the pan-Ethiopian identity of the previous imperial regime. This has exacerbated the political division between pan-ethnic and pan-Ethiopian nationalism. In the country’s political discourse, so-called dominant groups attempted to maintain their dominance through ethnic closure. The proponents of pan-Ethiopianism seek to create/restore the imagined and so-called Historic/greater Ethiopia by cultural homogenization and the elimination of alternative identities ( Mizan TV Center, 2021; OMN, 2021; Good Evening Ghana, 2021) and installing imperial/dictatorship ethnocracy. This could not go unopposed, resulting in the protests of the disadvantaged (or so they thought) ethnic groups toward the ethnic closure of the so-called dominant culture. This resulted in mutual ethnic polarization, as described in Ballard. For instance, Ballard (2002, p. 28) states that

The privileged groups routinely close ranks in ethnic terms to exclude their social subordinates in hegemonic patterns, so the excluded frequently respond by closing ranks themselves, the better to resist and subvert their subordination. When each side reacts in turn against the other, the outcome is often a rapid and escalating process of mutual ethnic polarization.

Such mutual ethnic polarization has been found in Ethiopian present and historical political debates. The current political squabbles and violent wars in Ethiopia are unquestionably the product of such an identity-based political division between pan-Ethiopian and pan-ethnic nationalists ( Zerai, 2017). Furthermore, the division is reflected by the contentious interpretation and misuse of ethnic-based federalism hosted by the EPRDF, which remains a major political issue in the country. The stark opposition to the multinational identity and federalism by the pan-Ethiopianist elite and scholars which claim the total dismantling of multinational federalism ( Lema, 2021; Atnafu, 2018). The main actors in this assume themselves as a state builders. The prominent group in this respect represents the elites of the Amhara ethnonational group.

For instance, the elites of Amhara claim that they are the builders of Ethiopia and hence, more than 90 percent of Ethiopia culture is Amharan calture. They insists that the dominance of the Amharan culture is the law of the nature and everyone need to accept this (see Amhara Media Corporation, 2018a and b). This is to mean that they aspire for the restoration of what consider the imperial regime’s restricted conception of Ethiopia’s cultural, social, political, and religious foundations, as well as its institutions, which failed to reflect the diversity that existed on the ground (see also Mekonnen, 1969). This claim of the Amharan elites is resemble with the what Belay (2013) termed as Amharization and chritianization In the post-199, the Amhara elites and scholars uses that the anti-Amhara discourses to wage anti-ethnonational political mobilization. For instance, Demerew (2024) argued that the mass violence has been committed on Amhara living in Oromia, Tigray and Benishangul Gumuz. Despite the Amharan elite consider themselves as state builder and pan-ethiopianist, the current trend signify the development of the ethnonational sentiment and the Amhara nationalism. This Amhara nationalism, however, is considered as a counter-nationalism to the alternative ethnonational identity politics ( Workneh, 2024).

With the provocation of elite urban Ahmara against ethnic based identity and federal structure, such claim has been revitalized and continued since 1991 as an anti-thesis to multicultural federalism, particularly to the Oromo nationalism which is aggravated and supported by military encroachment to the Oromia border and killing and displacing the Oromo from their land since 2018. For instance, as argued by Abebe (2023), the military intervention in the western Oromia with the participation of multiple actors (OLA, Fano and government) led to multiple civilian massacres on both Oromo and Amhara communities on the Oromia-Amhara borderland in 2022. In the same year an “estimated of 740,000 people were displaced within western Oromia and its borderlands with the Amhara and Benishangul-Gumuz regions” (ibid, p. 1). Abebe also argued that the conflict in the western Oromia is instigated by struggle for controlling territory and administration of the region which could be manifested by a contradiction and differing position between unitarist, federalist and Secessionists ideologies, and competing over national and regional power, particularly among the the elies of Tigray, Oromo and Amhara constituents. In this respect the conflict in and around Oromia, elsewhere in Ethiopia manifest the contestations between the proponents of pan-ethnic nationalism and Pan-Ethiopian nationalism, which is aggravated by the identity based claims and conflict over nature of the state structure, competition for monopolize power and resources sharing evidenced since the inception of federalism in Ethiopia.

In this regard, Pausewang (2005) clearly shows the resurgence of Pan-Ethiopianism as counter-nationalism to ethnic nationalism. He claimed that the urban Amhara, provoked by pressure to identify as an Amhara nationality, reverted to Pan-Ethiopian nationalism in post-1991. They strive to replace ethnic identities with a pan-Ethiopian national identity, suppressing or reforming any sub-national identity, and unifying all Ethiopians into a single identity, regardless of their ethnic origin. “Reorganizing Ethiopia as a federation of ethnically self-governing areas or states constituted a betrayal of Ethiopian unity, according to them. As a result, they accused the new government of a divide-and-rule strategy after 1991 (ibid, p. 280-281). Surprisingly, however, this group now reverted to particular identity politics, the Amharan nationalism, arguing that the pan-Ethiopian nationalism overshadowing the advancement of the Amharan nationalism. In this respect they made claims that almost all Ethiopian territory belongs to the Amhara and hence call themselves as Rist Asmelash (Restoration of territorial belongingness). For instance, Abebe (2023) argued that numbers of Amhara nationalists have made public statements claiming that Wollega is part of Amhara’s ancestral land and claim of its restoration. Similarly, there has been advocacy among Fano sympathizers to establish a committee – akin to the Wolkayt Tsegede Amhara Identity Restoration Committee – that would work to institutionally reclaim Wollega for the Amhara as a precondition for stopping Fanos from trying to take control of these territories by force. Hence this causes an irreconcilable conflict and contradiction between the Oromo and the Amharan elites and political factions.

Another claim and rationale for such supremacy is that victimization discourse that the Amhara were suppressed during the former EPRDF government, and ethnic cleansing and genocide were/are declared and perpetrated against Amhara due to their ethnicity ( Liyew, 2024). For example, certain political elites claim that the EPRDF government constructs and institutionalizes anti-Amharan institutions, policies, and systems with the primary goal of suppressing Amharan identity ( Amhara Media Corporation, 2018a and b). As a result, they claim for Amhara cultural dominance and the redesigning of state structure in order to reverse such oppression by restoring the imperial ideologies of the so-called real Ethiopians. For instance, despite no legal, policy and institutional formalities which selectively exclude and oppress the Amhara, scholars such as Messele And Ayalew (2025; p.12) clearly shows this victimization discourse saying that the advent of multinational federalism in Ethiopia “has led to the creation of laws that discriminate against the Amharas based on their identity.” In this regard, the former PP official and member of the parliament Gedu Andaragachew states that despite the Amhara people support the post-2018 political change, it suffer from its identity based indiscriminate massacre, displacement, imprisonment and humiliation ( Ethiopian Media Services (EMS), 2023). While he is one of a key supporter the military campaign against Tigray liberation front and Oromo Liberation Army, Gedu invariably opposed the state of emergence proclamation on Amhara political and military crises. The speech of Gedu clearly indicates the victimization discourses which is used to support the military and violent action of the Amhara political faction ፣ the Fano. He interpret the Fano military opposition as the resistance of the Amhara people which is born out from the comulative injustice ( የተጠራቀመ ግፍ ) againist Amhara.

Scholars such as Degu (2021) see the transformation of Amhara from pro-state to pro-ethnic political orientations undergone not in a manner that erased the prior commitment of Amharas to Ethiopian identity but as a new mode of opposing the ethnic-centered realm in neighboring regions. According to Chanie (2024), Amharas have endured many, widespread attacks from anti-Amhara political organizations such as the Oromo Liberation Front (OLF) and the Tigray People Liberation Front (TPLF), which formed a coalition and began hunting and killing ethnic Amharas shortly after the Derg regime fell and the Ethiopian People’s Revolutionary Democratic Front (EPRDF) came to power in 1991. Like the Serbians claim and blame of Tito regime, the elites of the Amhara, the militant Fano and diaspora claim as if the EPRDF policies and institutions are anti-Amhara and assuming the Oromo (perhaps all alternative identity politics outside the Amhara) as the ancient enemy of Amhara to wage war on the neighboring regions such as Oromia, Tigray and Benishangul Gumuz ( Chanie, 2024; Ethiopian Affairs, 2012; Institute for the Study of War, 2024) and engaging in resistances to government to protect Amhara from the existential threat ( Liyew, 2024) posed by the post-1991 politics of the country ( Kebede, 2025; Ethiopian Affairs, 2012) and the so assumed ancient and historical enemy of the Amhara people ( Ethiopian Affairs, 2012).

Another prominent political events that shows the unending dialectical contradiction between the competing nationalism is post-2018 political crisis and violent civil war in Tigray. According to Tofa et al. (2022, 6-7) the Tigray conflict is best explained “in terms of the contestations between a centralizing federal government and an autonomy-seeking, possibly secessionist, Tigray”. The conflcit in Tigray was instiggated by the substantial reduction of TPLF elites from dominating the political and administrative sphere of the country since 2018. this is particularly so following the refusal of TPLF to join the new coalition, prosperity party, which led to the re [lacing the TPLF cadre with independent Tigrayans and the TPLF saw that they are sidelined by the federal government ( Bethke, 2021; Abbink, 2024). In this respect, Bethke (2021, p. 3-4) states that;

The TPLF was ultimately also excluded from the central government, which was another tipping point in the escalation of the conflict. These policies of downgrading TPLF influence pursued by the Abiy administration eventually pushed the TPLF leaders to retreat to Tigray and mobilize for rebellion. With its origins as a victorious insurgent group and decades of dominating Ethiopian politics, TPLF leaders developed a sense of entitlement to state power. While other politically relevant groups perceived Abiys policies as leveling the playing field, the TPLF experienced the loss of relative power in the armed forces, ruling party and government as existential threats, and responded with mobilization and eventually violence.

Tofa et al. added that the Tigray conflict was also exacerbated by the contested claim made by Amhara and Tigray on a contested territory. The development of Amhara nationalism, with its demand for the reintegration of the two disputed areas between Tigray and Amhara into the Amhara region, intensified the tense situation. When the Amhara raised the topic of local self-determination in the Amhara-Tigray disputed territories, the TPLF repressed it, stating that the problem was one of governance rather than identity. The conflict between the Amhara political faction and the Tigray was minifested during the Tigray civil war in which the Amhara political faction support the federal government with aim of restoration of the certain territories from Tigray region, claiming that the territories are historically belongs to them. This manifests the ethnicization of both contested and uncontested territory by ethnonational groups evidenced and causes conflict not only in Tigray but also throughout the country.

There are widespread claims that ethnic/multinational federalism ^3^ in Ethiopia, which granted (at least in principle) the right to self-determination, self-expression, and the protection and recognition of ethnic-based identities, has never been a source of political conflict in the country as it was claimed ( Assefa, 2022). Pan-ethnic nationalists said that the difficulty in this respect is the lack of actual implementation of true multinational federalism and the EPRDF government’s unlawful reaction to constitutional questions. It is also worth noting the contributions of proponents of Pan-Ethiopian nationalism, who argued that the EPRDF/TPLF uses federalism as a means of divide and rule in the country’s present political discussions. Yusuf (2019a, b) clearly stated the paradox of ethnic federalism in Ethiopia, stating that ethnic federalism simultaneously empowers and disempowers ethnic groups. The upshot of this paradox is that ethnic federalism grants autonomous power (in theory) to regional authorities and empowers formerly excluded ethnic groups on the one hand, while party tentacles exercise powerful impacts and rigorous control over government structure on the other hand ( Zerai, 2017; Yusus, 2019a & b; Marzagora, 2017). Moreover, the federal structure dis-empowered the intra-regional minority equally. Marzagora (2017, p. 26) states that

The state-sponsored nationalism in Ethiopia of today (EPRDF era) thus draws both from the Great Tradition and from counter-historiographies. It attempts to accommodate and selectively emphasize one or the other. The contradictions between the two remain, and the new national ideology promoted by the EPRDF does not in any way represent a synthesis between the nations, nationalities, and peoples on one side and the imperial heritage on the other.

This, in turn, hampers the effective implementation of true and democratic federalism throughout the country, which later resulted in widespread political opposition from both Pan-ethnic nationalists and Pan-Ethiopian nationalists, who then sandwiched and disintegrated the EPRDF in 2018 ( Zerai, 2017; Yusus, 2019a & b). However, the contradiction and conflict between different ethnonational forces transformed into violent armed conflict ( Ishiyama, 2023) which is manifested by horizontal (between ethnonational group factions) and vertical (between government and ethnonational group faction) conflict ( ACCORD, 2025) which emerged and ignited in many parts of the country ( Birhan & Christopher, 2024). Actors in the violent conflict accusing one another arguing that the competing political faction and government committing genocide on their respective community. While neutral observatory bodies report the casualties from inclusive point of view, the factions and organization that assumed to be representing their ethno national groups commonly engaging in blaming one another for the sake of their interest (see for instance Amharan Association for Ammerica, 2022; Hailu, 2025; Hassen, 2022; Trueman, 2001; Addis, 2025). Hence, it is real that Ethiopia experiences violent political crises and conflict in many regions ( Abbink, 2024; Yihunie, 2024) which is repetitive and cyclical ( Abbink, 2024; Siyum, 2021).

**Figure 1.**
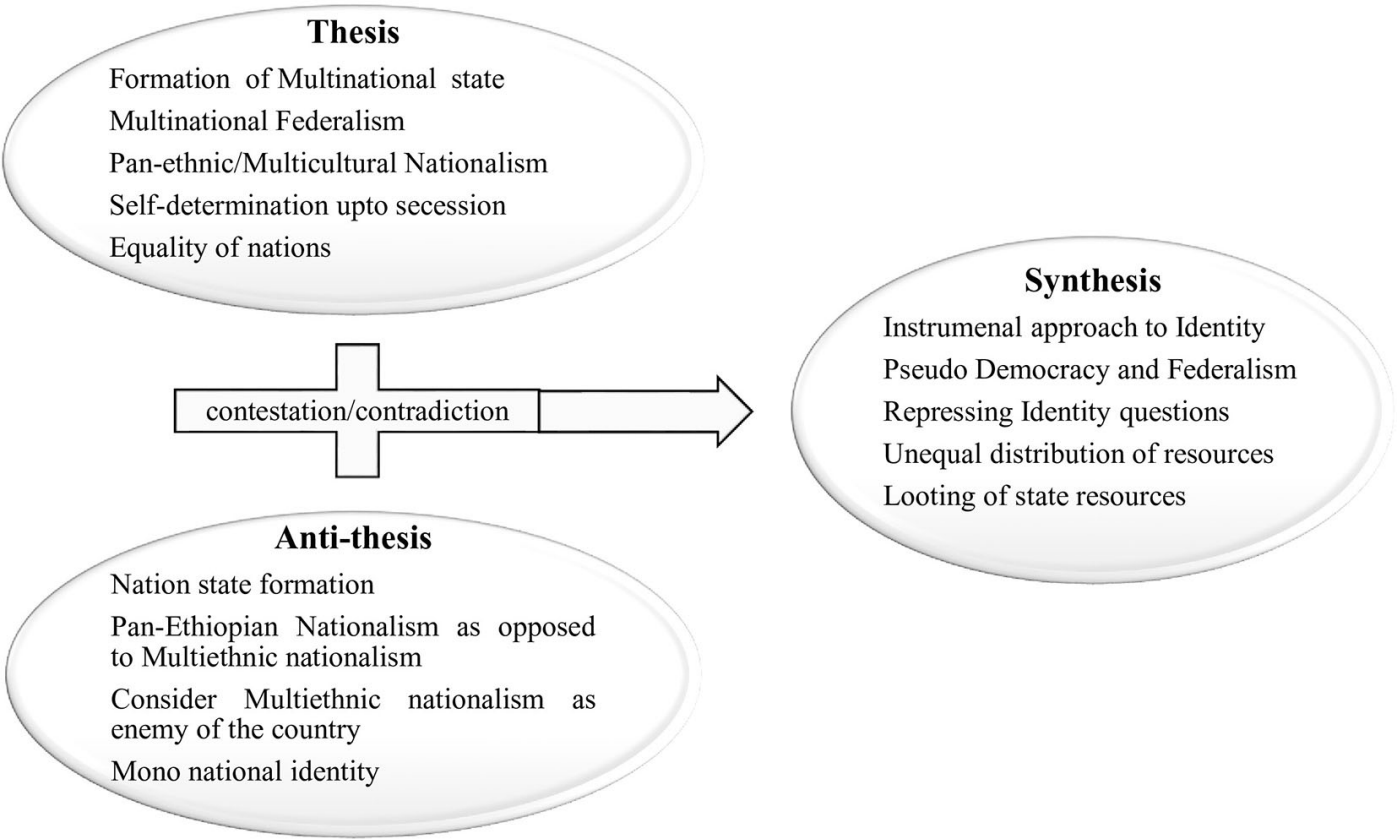
Dialectical model of Post-1991 Politics of Identity in Ethiopia. Note: Adapted from Assefa (2022) and supported by other literature (2024). The figure is adapted from previous publication which is free to adapt according the license policy of the journal license. Additionally, the previous figure is the design of the author.

Considering the dialectical process in light of this, there are still two contradicting and extreme questions regarding nationality and citizenship. These two dialectical contradiction are based on pan-Ethiopian nationalism and pan-ethnic nationalism which leads to the vicious cycle of political crises and violent conflict in Ethiopia ( Sloane, 2022). This dialectical process is shown in dialectical triads as follows ( Figure 1). The dialectical process of Ethiopian political history is always incomplete, considering the triads of dialectical process, thesis, anti-thesis, and synthesis. The historical and contemporary politics of the country indicate a vicious cycle of contestations, which is characterized by unending friction between thesis and anti-thesis ( Assefa, 2022). According to Yihune (2024), the successive regimes of the country since its inception as a modern state, failed to create a stable political system in Ethiopia. Yihune added that one of the account for the failure is the a conflict between ethnic groups (particularly Amhara, Oromo and Tigray) which emanated for the contending perspective and competing nationalism rooted in state making and remaking process ( Fiseha, 2024).

Based on the Hegelian dialectical triad shown in Figure 1, the post-1991 political contestations between competing nationalism can be represented by a dialectical model. The model was adopted from Assefa’s paper, “The Imperial Regimes as a Root of Current Ethnic-Based Conflicts in Ethiopia,” and it depicts dialectical contestations drive from the nation-state formation that took place pre-1991 ( Assefa, 2022). The reciprocal of Assefa’s model is used in this article to illustrate how the country’s post-1991 politics shifted from the imperial regime’s thesis to the anti-thesis, and vice versa. The pursuit of pan-ethnic nationalism has not been acknowledged by succeeding governments, according to Assefa’s model. During the 1960s and the 1970s, student revolution and aftermath, the quest for pan-ethnic nationalism attracted a wide range of individuals and political groups to challenge the narrow definition of Ethiopianism and to fulfill such pan-ethnic aspirations ( Mekonnen, 1969; Assefa, 2022; Horst, 2020; Marzagora, 2017).

However, since the 1991, the defeat of the Derg regime by a collusion force from diverse ethnic groups changed the country’s political space from pan-Ethiopian nationalism to pan-ethnic nationalism. The aspiration of one nation and one language of the imperial and Derg regime is replaced by the EPRDF regime that provides recognition (at least in principle) for the multinational identity, which is completely different from previous political regimes/systems ( Abbink, 2011). Most research concluded that ethnic identity was used as an instrument to perpetuate the political and economic benefits of political groups and elites of the EPRDF. Moreover, as in previous regimes, the EPRDF failed to create genuine and true multinational federal states. The persistence of multiple national identities and the repression of these identities by the imperial regimes were used as political opportunism by the EPRDF government to exercise divide and rule policy since its inception ( Assefa, 2022; Monenus, 2017; Adamu, 2013; McCracken, 2004).

The articulation of multinational politics (commonly used as ethnic politics in Ethiopia) during the EPRDF regime, like its predecessors, also failed to create a truly democratic federal state in Ethiopia. It failed to balance the competing identities in Ethiopia and remains fragile in nature ( Fiseha, 2007, 2012, 2018, 2019, 2022 and 2023). However, identity has been utilized as a means of perpetuating differences without creating a strong foundation for equality and democratic-based citizenship. While previous regimes openly repressed nationality questions, the EPRDF appropriated pseudo-identity-based politics to perpetuate differences and use it as a means of divide and rule. From an identity point of view, the EPRDF’s identity politics neither resolved the national oppression thesis nor created genuine and inclusive citizenship ( Assefa, 2022; Marzagora, 2017; Monenus, 2017; Adamu, 2013; McCracken, 2004). Hence, the pseudo multinational federalism of the EPRDF has been sandwiched between the proponents of pan-ethnic nationalism and pan-Ethiopian nationalism, which later made the regime unstable like the previous regimes.

Therefore, both the pre-1991 and post-1991 political history of Ethiopia signify that neither of the two extremes could be the solution for nationality questions and to create genuine and democratic citizenship. In this regard, Lidetu Ayalew argued that both the unitarist of the imperial and derg regime and the post-1991 dismissive politics have been tested and failed in the political history of the country ( Lemat, 2019). According to Lidetu, the implementation of both federalism and unitary state structure based on the existing diversity of the country. He did not totally reject the relevance of ethnic based politics in Ethiopia. However, he recommend the roles of ethnic based political parties to play their roles at local level (ibid). Despite the existence of constitutional provisions and policy frameworks to balance Unity (citizenship or civic nationalism) and diversity (ethnic nationalism) in Ethiopia, as provided in the FRDE constitution ( FDRE, 1995), the government of the EPRDF failed to sustain the balance because of structural and practical gaps in implementing the constitution ( Van der Beken, 2009). In this regard, Abbink (2011, p. 611) stated that despite the progress of the 20-year experiment, ethnic federalism in Ethiopia “neither compensate for the socio-political problems nor guarantee stability. At present, there still are constraints and dilemmas in the field of ethnicity and citizenship.” Moreover, Abbink (2011), Fiseha (2012, 2019, and 2023), and Belay (2013) testified to the failure of the EPRDF to genuinely implement federalism.

Moreover, the state and administrative structure was pseudo-federalist, as the central government dominated power and authority throughout the country. Agegnehu and Dibu (2016, p. 4843) concluded that “the development and consolidation of centralized dominant party rule which is paradox of genuine federalism, manipulates ethnic group in search of enlarging its power through applying a divide and rule approach.” As Keller and Omwami (2007) stated, the EPRDF government was not a true federalist and operated in a unitary state mentality. This means that multinational federalism in Ethiopia, during the EPRDF, was used as mask/sheepskin to mobilize community and consolidate power ( Assefa, 2022; Monenus, 2017; Adamu, 2013; McCracken, 2004).

Given its misappropriation and weakness in addressing the identity problems of the country, the post-1991 politics of Ethiopia indicates a complete shift from pan-Ethiopian nationalism to pan-ethnic nationalism. Viewed from the dialectical method, pan-ethnic nationalism became a thesis and pan-Ethiopian nationalism became an anti-thesis in the politics of the country. However, as in the previous regimes, this dialectic failed to create a strong and required synthesis. This means that, like the politics of its predecessors, the politics of the EPRDF regime failed to create genuine, democratic, and equality-based citizenship and failed to resolve historical problems through restorative justice. Such dialectical rotation of identity-based contradictions and opposing state formation approaches can be presented as shown in Figure 1. The figure represents the dialectical nature of Ethiopia’s identity politics in the post-1991 period.

## 6. Unending dialectics in Ethiopian politics

From the above analysis, it can be concluded that the processes of state formation, identity questions, self-determination, and ethnic conflict in Ethiopia are not only inevitable but also manifested by unending dialectical rotation. The current political situation indicates the unending dialectics of Ethiopian politics. The existing politics of ethnicity, identity, and citizenship indicate the unending process of political friction with the rotation of thesis and anti-thesis, which left the state-building projection of the country’s unstable and weak dialectical process. This was the result of the political miscalculation of the ruling regimes since the inception of modern Ethiopia which led to lack of mutual trust between government and ethnic identity groups in one hand and among ethnic identity groups in the other. That is, the wrong policy and political response of successive regimes to the nationality questions by successive governments resulted in a sustained and vicious circle of conflict ( Assefa, 2022; Jima, 2021). This makes the politics of the country a vicious circle of contradiction and conflict since its inception as a nation-state ( Jima, 2021; Zerai, 2017).

**Figure 2.**
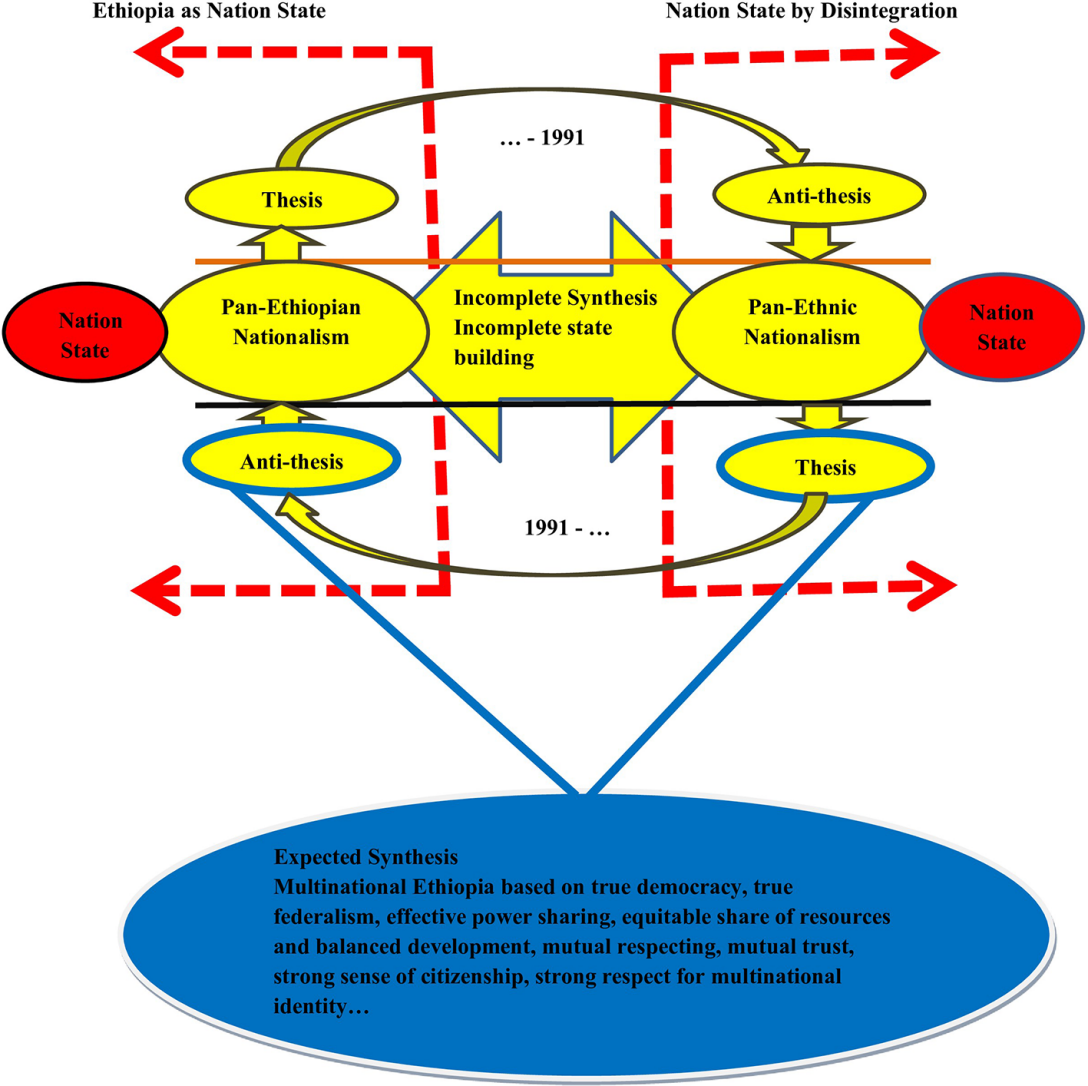
The cyclical Rotation of Thesis and Anti-thesis in Ethiopian Political History. Note: Developed by the author based on literature (2024) Keys to the Model a. Expected Synthesis: Aspiration of so many Ethiopians as general and politically active proponents of ethnicity-based identity. It is a required Ethiopia that was built based on consensus democracy, which was aspired by the majority of political elites (both pan-Ethiopians and pan-ethnic nationalists), and the community, which was inspired by security and peaceful coexistence. b. Incomplete Synthesis: This indicates incomplete state building attempted by successive regimes since the inception of the modern territory of the country as long as the contestations between the two nationalism led to violent vicious cycle of conflict. c. Nation State Through Homogenization: Top dawn approach to nation building and state formation based on one nation, one language, and mono-national identity. The model was attempted from Minilik II until the 1991. Such an attempt is termed as aggressive nationalism, which was attempted in European countries, such as France, Germany, and Italy. d. Nation State Through Disintegration: A reciprocal of “c” which aimed at forming single nation state through secession and breaking dawn the current Ethiopia into numbers of completely autonomous and sovereign states. e. Thesis/Anti-thesis: Dialectical triads that change with changes in the political history of the country. It was the only change in Ethiopian politics regarding identity. f. The Broken Red Arrow: Indicates the criticality of identity-based contestations (pan-Ethiopian and pan-ethnic nationalism). Currently, Ethiopia is at a critical stage, as the contradiction is followed by armed conflict. The arrow to the left indicates the extreme politics of Pan-Ethiopianists (nation-building through homogenization), while the arrow to the right indicates the politics of identity based on distinct national identity and the quest for independent political entities (formation of a nation state through disintegration and secession). g. The part of the model above the horizontal black line indicates the dialectical contradiction of the country’s pre-1991 politics. h. The part of the model below the horizontal orange line indicates the dialectical contradiction of the country’s post-1991 politics.

Figure 2 indicates the cyclical rotation of Ethiopian political history based on the dialectical model. The model combined the dialectical contradiction (contested political notion) of the imperial regime with that of the political nature of the country since the 1991 with specific reference to identity politics. This indicates how the pre-1991 political history (as indicated in Assefa, 2022, p. 112) of the country contradicted its post-1991. In the model, an attempt was made to indicate how current political conditions are linked to the country’s political history. The model indicates that the Ethiopian history of identity politics and contestations failed to create a strong and new synthesis that accommodates thesis and anti-thesis ( Assefa, 2022). The initial promises of the EPRDF were to create a multinational democratic state and ensure unity in diversity ( FDRE, 1995). In this regard, the late prime minister, Meles Zenawi clearly state that, in answering the question of journalist saying that “I am afraid that the ethnic federal structure would leads to disintegration of Ethiopia, if the benefit/advantage of being Ethiopian ( ኢትዮጵያዊነት ) weak no one will rebuild it. He stressed that “if every citizens has sufficient reasons to take/accept Ethiopian citizenship as proud, I do not expect that such danger will happen” Meles also stress the complementary relationship between diversity and unity saying that the advantage of unified as Ethiopia should not be contradicting with diversity and has its own economic, social, political and technological advantage. At the same time, Ethiopian unity is based on Ethiopian diversity and it should celebrate and recognize the existing diversity ( Aradom, 2017). However, practically the TPLF stretched its political tentacles to control and monopolize power, resulting in political exclusion, corruption, confiscation of national resources, utilization of identity for political and economic gain, and other multi-dimensional exclusions ( Jima, 2021; McCracken, 2004).

In this respect scholars such as Gedamu (2021), Teferi (2012), Ahadu and Abebe (2019) stress on the failure of ethiopian federalism by indicating some of the inherent problems in the federal design and its implementation. Gedamu argued that the failure of federal design in building democracy emanated from a clientelist relationship between federal and regional government. He also emphasizes the problem of the patrimonial clientelist relationship between levels of government that even extended to the public sphere through public bureaucracy. This, he argued, leads to the manipulation of federal design by the ruling party, which hampers democratization, self-administration, and minority representation. Moreover, monopolization of budget and financial disbursement also hampers the capacity of regional governments in exercising their power.

Important questions that need to be addressed at this point are the direction to which Ethiopia is moving: disintegration, nation building through homogenization, or building a Multinational state based on genuine democracy? The current political situation indicates a strong contradiction between Pan-Ethiopianism and Pan-ethnic nationalism. Compared to the historical process of the country, the current conflict and political discourses are not in position to create the expected synthesis, as shown in Figure 2. This chaos and political crisis lead the future fate of country to be indeterminate at the current time. The country is at the bottom of multiple crises and chaos, with no clear political roadmap and vision regarding how to resolve the current political contestations. There is a conflicting vision of the country among competing political actors ( France 24, 2021).

Hence, the history of the country is highly articulated at the current time in order to justify violent war and restoration of domination in post-1991 and aggravated since 2018. More extremely, the shift in the discourse of creating multicultural Ethiopia to the extreme narration of secessionist discourse is another indication for the changing trajectory of identity based political narratives. This aspiration has been intensified in the political transition of the country since the 2018 and the quest for ethnicization of both contested and uncontested territory by the faction of specific ethnic group. In the words of Semir Yusuf, the incident and intensity of conflict has surged since the political liberalization of 2018 ( ISSAfricaTV, 2019). According to Yusuf, one of the the major reasons for this is the increasing ethnic mobilization which is manifested by the ascendant Oromo, Amhara, Tigray and other ethnic mobilization which is antagonistic to one another in same ways (ibid). These ethnic mobilization are now supported by military interlude to control some territory by conquest and extinction of the indigenous people in some marked area of Wollega, Shewa, Tigray, Amhara, Benishangul Gumuz and the rest of the country by the ethnocratic factions assumed to be representing ethnic groups which resulted in conflict and civilian fatalities ( News Analytica, 2022; Al Jazeera English, 2021).

The post-2018 political transition signified by high level contestations between the competing nationalism which is emanated from the aspiration of incumbent governments and political faction from diverse identity groups. The first resistance to government ignited with the so assumed plan of government restoration of the unitary form of government. This resistance was first exploded from Oromo political faction (prominently OLF) following the death of Hacalu Hundessa, and later from Tigray (from TPLF) and Amhara (from Fano). The current incumbent government faces challenge from both the pan-Ethiopian and pan-ethnic nationalism. While the government claim that there should be a balance between diversity and unity, the opposing political factions accuse the government from two competing and conflicting nationalism. One from pan-ethnic nationalism which claim that the existing government failed to resolve the nationality question. Such resistance come from those that claim more and more of autonomous self-government and self-determination. The Oromo and the Tigray political factions are the prominent resistances to the central government which defined by ethnonational identity politics.

The second challenge emanated from, more or less, the so called Pan-Ethiopianist elite. Some of these elites accuse the post-1991 regime as the anti-Ethiopian and divisive regime. Most of the Amharan political elites and nationalist and some on the middle of the road elites aspire for the restoration the pre-1974 politics and nation building project. They consider the post-1991 politics as the politics of the country as the politics of the Tigray regime and the post-2018 as the politics of the Oromo regime that imposed on Ethiopia in general and the Amhara people in particular ( Brussels Press Club TV, 2021). The resistance could be seen in various ways. Some claim that the ethno-national politics was manipulated for the benefit of the Tigray political elites and against the Amhara people (ibid). There are also a claim based on the ownership of the regional state as stipulated in the FDRE constitution and the constitutions of the regional states.

Some elites misinterpret the regional constitutional based on the categorization of the titular and non-titular of the regional residents. For instance, the regional constitutions provide the ultimate power and authority of the region to the regional ethnic groups is misinterpreted as if the other ethnic groups are not permitted to live and own resources in other region, and to take part in the political and administrative works of other region. For instance, despite the Oromia National Regional State Constitution state that the Oromo people is the owner of the sovereign authority in the region, the subsequent provisions of the constitution provide social, political and economic right of every person that resides in the region. While the regional constitutions are almost has the same provisions in their content, the Amharan elites misinterpret the regional constitutions as if it is anti-Amhara constitutions. For instance the Oromia and the Amhara regional constitutions article 38 express the same thing with different terminology. Oromia regional constitution reads as every person that live in the region while the Amhara regional constitution reads as every Ethiopians has a right to vote and to be voted, to take part in any government institution without any discrimination based on race, language, ethnicity, religion, political opinion, sex and other status (for more see The Council of Amhara National Regional State, 2001 and Chafe Oromia, 2001). Hence, the regional constitutions provide equality to each and every person that lives in the region. A close and comprehensive look at the constitutions provide personal, groups, economic, social, political, democratic and human right.

Other challenge manifested by political competition to hold a grip on the central government by the prominent political elites of the specific ethno-national group. In the post-1991 the regime was accused to be the government of Tigray while the post-2018 regime is accused as if it is the government the Oromo (selectively assumed to be the government of the Oromo Liberation Front (OLF)). In this regard there has been the “ *Bale Gize*” discourse used by some Ethiopianist elites to indicate that the current government of the country is the government of the Oromo and the Oromo is the “ *Bale Gize”.* Such discourse indicate the competition between ethno-national groups to take a control of the key position in the central government (particularly the prime minister position) knowing that holding the position is the means to consolidate power and means of resource distribution and redistribution.

The more critical challenge at the current time is the change in the trajectory of pan-Ethiopian nationalism into ethno-religious nationalism by extreme Ethiopianists. Moreover, the current issue of religious institutions poses additional fuel to identity-based contradictions in the country. The aim of the discourse of ethno-religious nationalism is the restoration of the greatness and dominance of specific ethno-religious groups, the so-called great tradition of imperial regimes (the imperial ethnocracy). They argued that the greatness and dominance of such ethno-religious was lost with the demise of the imperial regime in 1974 (see Amhara Media Corporation, 2018a and b). The marriage between the religious elite and political elites at the current time changed the nature of political discourse in the country, which might be a critical juncture for religious contradiction, conflict, and additional political ferment. Such a changing trajectory might exacerbate the historical and contemporary political crisis, adding new fuel to the existing problem of identity politics.

## 7. Implications

The history of Ethiopia indicates that the majority of political contestations of the country constitute identity (ethnicity) dimensions. Conflict of resource ownership, history of the country itself, political polarization, questions of political participation and representation, government responses to political questions, state formation and state-building projects, and other government decisions are seen from identity (ethnicity) perspectives and contestation therein. The country is still sandwiched between extreme identity-based political discourses, which are a source of major political problems in the country. The country is sandwiched between two extreme nationalism (including both pan-Ethiopian nationalism and pan-ethnic nationalism) because of the failure of considering the complementary of both nationalism as conceptualized by Parekh and Laclau. The contradiction between the two nationalisms has been ignited by the claim to attain and monopolize the central power of the country and ownership of resources in the name of specific ethnic groups. This is implied by the unending political competition among the elites of ethnonational groups.

This makes the politics of a country prone to a vicious circle of contradiction and conflict, which hampers desirable state formation and building. Hence, the country currently has three political options: the formation of a nation-state through homogenization, the formation of a nation-state through disintegration/forceful dissolution, and the formation of a democratic multinational state. Now, some of the political discourses of so-called pan-Ethiopian nationalism have changed to ethno-religious nationalism, in which some elite’s quest for ethno-religious dominance in the politics of the country. Numerous pseudo-pan-Ethiopian nationalists call upon religious, political, and ethnic dominance in the country. Still, there are numerous political elites in the middle of the road, which gave ample opportunities to create a consensus based multinational state.

This further implies that Ethiopian history and the current political landscape indicate that the prescription of one side of nationalism at the expense of the other will never resolve the political crisis and a vicious circle of contradiction in the process of building strong and inclusive state institutions. Ethiopians’ historical and ongoing crises are rooted in the history and process of creating and remaking Ethiopian polity. Professor Merera calls this the transition from crisis to crisis through crisis. In Assefa (2022), this crisis is called a vicious circle of conflict, and Jima (2021) in his article, Vicious circle of Ethiopian politics: Prospects and challenges of current political reform, he calls this a vicious circle of Ethiopian politics. McCracken (2004) calls Ethiopian history crisis history. This indicates that Ethiopia has inherited political crises throughout its history, which is characterized by an unending dialectical contradiction between pan-Ethiopian nationalism and pan-ethnic nationalism.

This implies that the application of ethnocratic politics in state making and polity building will never end the contradiction between the competing nationalism but it will aggravate the contradiction and create violent conflict. Furthermore, the vicious cycle of contradiction between ethnonational identity and civic identity in Ethiopia indicates the political and institutional failures to consider the two nationalisms as complementary in its nature. The successive regimes of the country quests for implementing one nationalism at the costs of the other in setting legal and institutional frameworks. This later indicates the application of vicious cycle of dialectical contradiction which further implies the application of the Hegelian dialectical method to indicate the rotation of thesis and antithesis which failed to evolved to synthesis. Hence, the Ethiopian identity politics and history indicate unending dialectic rather than the linear and progressive historical development as indicated in the Hegelian dialectical approach.

## 8. Recommendations

Therefore, a balanced view of identity based political discourses, claims and problems in the country is a primary condition to be nurtured in the political discourse of the country, if one needs to reduce the actual and potential ethnic conflict in Ethiopia. In particular, considering the current political discourse of the country, it is recommended that the existence, recognition, and institutionalization of both identities is a prerequisite to reverse the conflicting history of the country and ensure peaceful coexistence for the current and future generations.

Scholars of identity politics recommend that there should be a distinction between nationality and citizenship. Given the subtlety of the distinction between nationality and citizenship, such a separation has important implications in resolving identity problems that emanate from a faulty definition and misinterpretation of identity in Ethiopia. As a multinational state, there should be coexistence of civic identity (citizenship) and national identity (Nationality) in Ethiopia. Hence, there should be a genuine definition of identity that could reverse the antagonistic relationship between the pan-Ethiopian (faulty consider Ethiopia as a nation) and pan-ethnic proponents. Hence, mutual recognition of both citizenship and nationalism is recommended. Regarding this Piattoeva (2016: np) conclude that “Citizenship must now reconcile the initial pursuit of equality and universality with the recognition of difference. This question is essentially about whether the two concepts can be perceived in a multilayered fashion, as opposed to viewing them through a zero-sum game logic.”

Dalgliesh (2013) also identifies important preconditions in which the aporia on identity-based discourses could be reduced. These mechanisms constitute legitimacy, representation, and authority. By legitimacy, Dalgliesh means that identity-based discourse needs to widen the bases of informing political agendas. Representation indicates the improvement of the number of groups that participates in the policy-making process. While authority represents the recognition of an individual’s choice of identity without imposing what is determined to be true identity produced elsewhere by knowledge and expertise (ibid). Therefore, identity politics to be seen from an optimistic point of view and lead to positive social transformation, needs the fulfillment of the above three preconditions. Hence, the identity politics which is failed to legitimately recognize alternative identity, failed to improve representation/participation of group members in policy making process and imposes the pre-identified and defined identity on the rest of the groups will leads to divisive and violent conflict.

What Ethiopia needs is, therefore, the creation and strengthening of democratic rules, institutions, the environment, and policies in which one’s own identity can be positively and equally treated without infringing on others. There should be mechanisms that prevent the forceful attempt to impose one’s own identity or the identity that one likes over another, because no one is the maker of identity for another. This is because democracy needs free human choice with which individuals make their own identity choice and enjoy identity rights without infringing on others. A thoughtful and wise political discourse is needed to manage identity tension among elites in the country. In particular, considering the current political situation of the country, one should not carelessly repress claims for representation, self-determination, and self-administration, and quests for identity recognition raised based on multinational identity.

Both historical and current political situations in Ethiopia indicate that Ethiopia is a multicultural state in which more than 80 (87 nations according to research conducted by the Ethiopian Nationality Study Institute in 1987) nations persist. Not only persists but also indicates the impossibility of assimilating/repressing these multinational identities along the narrow definition of Ethiopian nationalism. Ethiopian nationalism ( የኢትዮጵያ ብሄርተኝነት ) is a paradoxically created identity by the political elites of the so-called dominant culture/group of the imperial regimes. The historical and current political problems of the country have emanated from an attempt to impose narrowly defined nationalism. Hence, the application of a more complete dialectical approach in dealing with the antagonism between the proponents of Ethiopian nationalism and multicultural nationalism is also important for harmonious coexistence and successful state-building. As shown in Figure 2, it is recommended that the expected synthesis, which is detached from the unending dialectical contradiction between Pan-Ethiopianism and Pan-ethnic nationalism, should be Greater Ethiopia, in which both citizenship and nationalism are respected based on mutual understanding and belongingness.

To this end, well-known scholars of federalism, Fiseha (2023), recommend the application of both integrative and accommodative federalism to balance the two extreme quests, integration for pan-Ethiopian nationalism, and accommodation for pan-ethnic nationalism in the country. Assefa and Wami (2023) recommend the realization of genuine and real multicultural democratic federalism rather than mobilizing citizens from an instrumental point of view.

Moreover, the complimentary method of the Switzerland federation should also be implemented to protect the rights of intra-regional minorities, which Ethiopian federalism practically failed to protect. Switzerland could be the best example where cantonal-level minorities are protected without contravene cantonal autonomy and self-rule. According to Belser (2018), the European Framework Convention and the new framework for protecting national minorities have been taken seriously by Switzerland, without curtailing cantonal and municipal authority. The idea of national minorities has not been utilized to replace established methods of accommodating minorities but rather to enhance them. Therefore, Ethiopian need to install some legal and institutional design to protect the right of intra-regional minorities.

## Data Availability

The data used in this research were obtained from secondary sources. Raw data was not collected for and used in this particular article.

## Footnotes

^1^
Identity politics is defined as the intentional/deliberate use of identity discourse for political purposes, with the goal of addressing the quest for power sharing and autonomous self-government by converting multiple preferences into policy and channeling them into political institutions, resulting in an unavoidable and morally charged experience that everyone goes through during the political process.

^2^
Dialectic is a philosophy that stresses the conceptualization of opposing conceptions, ideas, and views in order to demonstrate the evolution and development of concept, idea, and opinion from contradiction and it may be applied to a wide range of subjects. Dialectics is a philosophical argumentation strategy that comprises a conflicting process between competing points of view for more (see The Editors of Encyclopaedia Britannica, 2025; Stanford Encyclopedia of Philosophy, 2020; Berto, 2007; Khan, 2008).

^3^
Ethnic/multinational federalism is the form of governance in which the structure of the state is designed based on ethnic identity politics where each major territorially organized ethnic group are given political autonomy in administering their affairs. In this respect multinational federalism constitutes a federated states/regions/provinces in which minority group become a majority in their respective state/regions/provinces (for more see Burgess, 2012; Seymour and Gagnon, 2012; Gagnon, 2021). This is why scholars such as Gagnon argued that multinational federalism is the manifestation of the advancement of nations within a territorially confined state. It recognizes the presence of multiple identities in a polity.
