# Supplementary material for: Unending dialectical politics of identity in Ethiopia
Source: F1000Res. 2025 Dec 27;14:137. Originally published 2025 Jan 27. [Version 2] doi: 10.12688/f1000research.160666.2 (PMC12375204; doi:10.12688/f1000research.160666.2)
Supplement: Peer review history

# Reviewer report: Reviewer response for version 1

**DOI:** 10.5256/f1000research.176597.r394546

**Published:** 2025-08-28

**Authors:** Debora Valentina Malito

**Affiliations:** 1 Xi’an Jiaotong-Liverpool University, Suzhou, China

This paper offers an insightful exploration of the ongoing dialectical tensions between national and ethnic identities in Ethiopia. It contributes to the political theory of the state in Africa and has the potential to shed light on the underlying conflicts associated with contemporary ethnic federalism and the war in Tigray. I have three main observations:

 First, the discussion on dialectical methodology and its foundations is somewhat limited. It would be valuable to acknowledge Hegel’s framework limitations, as highlighted by postcolonial critiques. For instance, Fanon (1952, Black Skin, White Masks) emphasizes the need for ruptures rather than synthesis, and Hegel’s master-slave dialectic notably overlooks actual anti-slavery revolts (see Buck-Morss 2009, *Hegel, Haiti and Universal History*). Hegel’s system tends to absorb contradictions into synthesis, neglecting how structural violence creates and perpetuates irreconcilable antagonisms. Acknowledging the value of post-colonial and post-Hegelian dialectics could strengthen the argument (see Laura Doyle, 2020, *Inter-imperiality*; Laura Doyle et al., 2025, *Decolonial Constellations*). While it is possible that this dialectical approach has been addressed in another published paper, discussing post-Hegelian dialectical methodology here could help address some limitations, as the attention to the dynamic interaction between thesis and antithesis.

 Second, the article refers to the tendency to preserve dominant cultures but does not closely define the inter-ethnic relations among the major groups (i.e. Amhara, Tigray, Oromo, and Somali) that have shaped Ethiopian state formation and its contestations. To clarify the theoretical argument, it would be beneficial to provide concrete historical examples where the “vicious circle of contradiction and conflict” is evident.

 Third, the analytical rigour and contribution of the dialectical lens is not always clear. In this context, the authors seem to interpret dialectics more as a vicious circle than a virtuous one. It is important to note that dialectical ontology should not be solely equated with the negative aspects of this process. This leads me to critique the conceptualization of synthesis as the endpoint of contradiction rather than recognizing its mutual constitution and intersubjectivity (as the postcolonial critique does).  A more grounded empirical analysis would also help to shed light on the theorized argument.

Is the work clearly and accurately presented and does it cite the current literature?

Yes

If applicable, is the statistical analysis and its interpretation appropriate?

Not applicable

Are all the source data underlying the results available to ensure full reproducibility?

Yes

Is the study design appropriate and is the work technically sound?

Partly

Are the conclusions drawn adequately supported by the results?

Yes

Are sufficient details of methods and analysis provided to allow replication by others?

Partly

Reviewer Expertise:

Critical International Relations; Africa's International Relations; political theory of state formation and sovereignty

I confirm that I have read this submission and believe that I have an appropriate level of expertise to confirm that it is of an acceptable scientific standard, however I have significant reservations, as outlined above.

# Response

**Published:** 2025-08-28

**Authors:** Tefera Asefa

Dear Debora Valentina Malito,

 Thank you very much for your interesting comments and observation. I will address the commwnts as much as possible.

# Response

**Published:** 2025-11-17

**Authors:** Tefera Asefa

**Observation 1:** First, the discussion on dialectical methodology and its foundations is somewhat limited. It would be valuable to acknowledge Hegel’s framework limitations, as highlighted by postcolonial critiques. For instance, Fanon (1952, Black Skin, White Masks) emphasizes the need for ruptures rather than synthesis, and Hegel’s master-slave dialectic notably overlooks actual anti-slavery revolts (see Buck-Morss 2009, *Hegel, Haiti and Universal History*). Hegel’s system tends to absorb contradictions into synthesis, neglecting how structural violence creates and perpetuates irreconcilable antagonisms. Acknowledging the value of post-colonial and post-Hegelian dialectics could strengthen the argument (see Laura Doyle, 2020, *Inter-imperiality*; Laura Doyle et al., 2025, *Decolonial Constellations*). While it is possible that this dialectical approach has been addressed in another published paper, discussing post-Hegelian dialectical methodology here could help address some limitations, as the attention to the dynamic interaction between thesis and antithesis.

**Response 1:** Further discussion of dialectical methodology is added in the theoretical framework of the revised article. Despite the some of the literature recommended by the reviewer are important, the issue under investigation could be sufficiently addressed using Hegelian dialectical triad. But the Hegelian dialectical triad is used to indicate the cyclical rotation of contradiction between the two nationalisms in Ethiopia (for more see the last paragraph of theoretical framework). I hope the indicated literatures will be a valuable source for my future research works.

**Observation 2:** Second, the article refers to the tendency to preserve dominant cultures but does not closely define the inter-ethnic relations among the major groups (i.e. Amhara, Tigray, Oromo, and Somali) that have shaped Ethiopian state formation and its contestations. To clarify the theoretical argument, it would be beneficial to provide concrete historical examples where the “vicious circle of contradiction and conflict” is evident.

**Response 2:** In the revised article, the contradiction between these major ethnic groups and its ramifications has been included. Certain practical evidence and events that show the unending dialectical contradiction are also included in the revised article. Violent conflict that emanated from these contradictions is also included in the revised article.

**Observation 3: **Third, the analytical rigor and contribution of the dialectical lens is not always clear. In this context, the authors seem to interpret dialectics more as a vicious circle than a virtuous one. It is important to note that dialectical ontology should not be solely equated with the negative aspects of this process. This leads me to critique the conceptualization of synthesis as the endpoint of contradiction rather than recognizing its mutual constitution and intersubjectivity (as the postcolonial critique does).  A more grounded empirical analysis would also help to shed light on the theorized argument.

**Response 3:** The issue of dialectical theory and its application in Ethiopia is added under the new topic (theoretical framework). I hope this address the comment as much as possible. For grounded empirical analysis, some cases and events that shows the political crises in Ethiopia have been added in the revised article.

# Reviewer report: Reviewer response for version 1

**DOI:** 10.5256/f1000research.176597.r396051

**Published:** 2025-08-23

**Authors:** Usman Ayegba

**Affiliations:** 1 University of Gambia, Serekunda, The Gambia

REVIEW REPORT ON THE PAPER: “UNENDING DIALECTICAL POLITICS OF IDENTITY IN ETHIOPIA”

 Article’s Summary:

 No doubt, the article explores the persistent and evolving nature of identity politics in Ethiopia, emphasizing the dialectical tensions between ethnicity, state formation, and democratic governance. Drawing from historical, political, and philosophical perspectives, it examines how the Ethiopian People’s Revolutionary Democratic Front (EPRDF)’s ethnic federalism institutionalized identity as the primary mode of political representation and contestation.

 The paper clearly argues that while the 1995 Constitution aimed to empower historically marginalized ethnic groups through self-determination, the federal arrangement inadvertently deepened divisions, incentivized ethno-nationalist mobilization, and delegitimized pan-Ethiopian unity. It also analyzes the post-2018 reformist momentum under the current leadership of Prime Minister Abiy Ahmed, which challenged the hegemony of ethnic politics but also led to political backlash, inter-ethnic violence, and renewed calls for autonomy or secession by regional actors.

 Thus, the article adopts a dialectical theoretical scholarship or lens, perhaps, mainly influenced by Hegelian-Marxist thought, to frame identity politics as a recursive, contradictory process where state attempts at resolution give rise to new contradictions. It concludes that Ethiopia’s political trajectory is trapped in an unending dialectic of fragmentation and unification, with neither a return to centralized nationalism nor a stable ethnic federalism providing sustainable solutions.

 Paper Evaluation Based on Certain Criteria

 1. Is the research question clearly stated and significant?

 Partly.

 Strength

 The paper and/or article touches on a timely and important topic with profound implications for peace, state-building, and democratic governance in Ethiopia and Africa more broadly.

 Limitation

 Despite the obvious strength of the paper, it is not without limitation. The research question is not clearly articulated as a focused inquiry. Theoretical ambition and narrative breadth may overshadow a clear hypothesis or guiding question.

 Recommendation

 The author should reframe the research question explicitly at the beginning. For example: *“How has Ethiopia’s ethnic federalism contributed to the persistence of identity-based political conflicts, and what dialectical tensions does this reveal in state-ethnicity relations?”*

 2. Is the theoretical framework appropriate and clearly applied?

 Partly.

 Strength

 The use of dialectical theory offers an innovative lens for interpreting Ethiopia’s complex political dynamics.

 Limitation

 The theoretical underpinnings are vaguely referenced and insufficiently operationalized. The dialectic is more metaphorical than analytically rigorous.

 Recommendation

 The author needs to clarify the dialectical framework whether, as used in the context of the paper, it is derived from Hegelian, Marxist, or postcolonial dialectics. Also, effort should be made in defining the triadic movement (thesis-antithesis-synthesis) and show how it maps onto historical or contemporary events in Ethiopia. Incorporate political theorists like Mahmood Mamdani or Benedict Anderson to enrich the conceptual foundation.

 3. Is the methodology sound and clearly described?

 No.

 Limitation

 The article lacks methodological transparency. It is unclear whether it is based on primary data, discourse analysis, historical interpretation, or case studies.

 Recommendation

 Specify the method vis-à-vis:

1. If using qualitative analysis, describe data sources (e.g., government documents, speeches, interviews).
2. If historical analysis, clarify the temporal scope and sources consulted.
3. If discourse analysis, outline the corpus and coding approach.

This section is crucial for scientific rigor.

 4. Is the analysis compelling and well-supported by evidence?

 Partly.

 Strength

 The paper offers clearer interpretations of Ethiopia’s political history, especially in linking identity politics with constitutional arrangements.

 Limitation

 There is insufficient engagement with empirical evidence to substantiate claims. The article is more interpretive than analytic.

 Recommendation:

 Provide concrete examples (e.g., Tigray conflict, Oromia protests, Amhara claims) with references to policy documents, statistics, or fieldwork. Ground claims in data to avoid sweeping generalizations.

 5. Does the article contribute new knowledge or perspectives?

 Obviously, yes.

 Strength

 The dialectical framing of Ethiopia’s identity politics is intellectually original and offers an alternative to more static ethnic conflict models.

 Recommendation

 The originality of the conceptual approach is commendable. However, its contribution would be clearer if the theoretical novelty were better structured and substantiated with empirical grounding.

 6. Is the writing clear, coherent, and well-organized?

 Partly.

 Strength

 The prose is conceptually ambitious and intellectually rich.

 Limitation

 It occasionally veers into dense abstraction without sufficient explanation for non-specialist readers.

 Recommendation:

 Authors should do well by defining key terms early – terms or concepts like *dialectics, ethnic federalism, identity politics*. Besides, use subheadings to improve structure, and also reduce jargon and overly philosophical passages unless directly tied to the argument.

 Constructive Suggestions and Must-Address Points

 To make the paper scientifically sound, the following must be urgently addressed by the authors:

1. Clarify the research question and link it directly to the theoretical and empirical inquiry.
2. Define and apply the dialectical framework rigorously, not just as a metaphor but as an analytic tool.
3. Specify the methodology and provide transparency about sources and analytical procedures.
4. Include more empirical data or case illustrations to support arguments.
5. Improve structure and accessibility, including clearer transitions, key term definitions, and logical flow of ideas.

CONCLUSION AND RECOMMENDATION:

 The paper presents an important and thought-provoking argument with real potential to advance debates on identity politics in Africa. However, to meet the standards of scientific rigor and clarity, the author must strengthen the theoretical framework, methodological transparency, and empirical grounding. Nonetheless, on the recommendation, major revisions required!

Is the work clearly and accurately presented and does it cite the current literature?

Partly

If applicable, is the statistical analysis and its interpretation appropriate?

Partly

Are all the source data underlying the results available to ensure full reproducibility?

Partly

Is the study design appropriate and is the work technically sound?

Partly

Are the conclusions drawn adequately supported by the results?

Partly

Are sufficient details of methods and analysis provided to allow replication by others?

Partly

Reviewer Expertise:

Political Science, International Relations and Diplomacy, and African Politics

I confirm that I have read this submission and believe that I have an appropriate level of expertise to confirm that it is of an acceptable scientific standard.

# Response

**Published:** 2025-08-28

**Authors:** Tefera Asefa

Dear Usman Ayegba,

 thanks a lot for you time and efforts to provide constructive commenents. I will add your comments in the revised version of the manuscript.

# Response

**Published:** 2025-11-17

**Authors:** Tefera Asefa

**Recommendation 1:** The author should reframe the research question explicitly at the beginning. For example: “How has Ethiopia’s ethnic federalism contributed to the persistence of identity-based political conflicts, and what dialectical tensions does this reveal in state-ethnicity relations?”

**Response 1:** Thanks for reminding me to set research question for the article. As per the comment research question is framed and included in the revised article. For more see introduction part the last paragraph.

**Recommendation 2:** The author needs to clarify the dialectical framework whether, as used in the context of the paper, it is derived from Hegelian, Marxist, or postcolonial dialectics. Also, effort should be made in defining the triadic movement (thesis-antithesis-synthesis) nd show how it maps onto historical or contemporary events in Ethiopia. Incorporate political theorists like Mahmood Mamdani or Benedict Anderson to enrich the conceptual foundation.

**Response 2: **This comment is also addressed with a brief theoretical framework added as new topic to the manuscript. However, Mohammed Mandani and Benedict Anderson are most appropriate for my manuscript that I am now preparing for future publication. Hence, I hope the reviewer would understand this.

**Recommendation 3:** Specify the method vis-à-vis:If using qualitative analysis, describe data sources (e.g., government documents, speeches, interviews). If historical analysis, clarify the temporal scope and sources consulted. If discourse analysis, outline the corpus and coding approach. This section is crucial for scientific rigor.

**Response 3:** Thanks for the methodological comment. Some changes have been made to the methods and material part of the article to address the comment. In this part the sources of the information, collection method and analysis method is indicted briefly.

**Recommendation 4**: Provide concrete examples (e.g., Tigray conflict, Oromia protests, Amhara claims) with references to policy documents, statistics, or fieldwork. Ground claims in data to avoid sweeping generalizations.

**Response 4:** Thanks for indicating to use empirical evidence in the article. Based on the comment, some important empirical evidence from different sources was added to indicate the practical results of the political contradictions. These changes are made in numerous parts of the article, but prominently in part 5 and 6 of the article shows these changes.

**Recommendation 5:** The originality of the conceptual approach is commendable. However, its contribution would be clearer if the theoretical novelty were better structured and substantiated with empirical grounding.    

**Response 5:** I hope the response to comment 2 and 4 are also work for this comment.

**Recommendation 6:** Authors should do well by defining key terms early – terms or concepts like dialectics, ethnic federalism, and identity politics. Besides, use subheadings to improve structure, and also reduce jargon and overly philosophical passages unless directly tied to the argument.

**Response 6:** The recommendation is very relevant and definitions of some terminologies are added as endnote in the revised article.

# Reviewer report: Reviewer response for version 1

**DOI:** 10.5256/f1000research.176597.r389733

**Published:** 2025-07-25

**Authors:** Al Chukwuma Okoli

**Affiliations:** 1 Federal University of Lafia, Lafia, Nigeria

The paper is well conceived, developed and structured. It engages its subject matter quite well. However, it would have a lot better if a brief political history of Ethiopia is added after the introduction.

 Also, the implications of the study's submission for both theory and practice need to be more amply and clearly highlighted.

 The paper holds immense scholarly merit.

Is the work clearly and accurately presented and does it cite the current literature?

Yes

If applicable, is the statistical analysis and its interpretation appropriate?

Not applicable

Are all the source data underlying the results available to ensure full reproducibility?

Yes

Is the study design appropriate and is the work technically sound?

Yes

Are the conclusions drawn adequately supported by the results?

Partly

Are sufficient details of methods and analysis provided to allow replication by others?

Yes

Reviewer Expertise:

Security Studies, Intergroup Relations

I confirm that I have read this submission and believe that I have an appropriate level of expertise to confirm that it is of an acceptable scientific standard.

# Response

**Published:** 2025-07-26

**Authors:** Tefera Asefa

Dear reviewer,

 Thank you for your insightful comments and I will add the suggestions provided in the new version of submission.

# Response

**Published:** 2025-11-17

**Authors:** Tefera Asefa

**Comment 1**: It would have a lot better if a brief political history of Ethiopia is added after the introduction.

**Response:** Thanks for the comment. The comment is accepted and included in the revised article.

**Comment 2:** Also, the implications of the study's submission for both theory and practice need to be more amply and clearly highlighted.

**Response:** For the purpose of this comment, the theoretical framework is added, and the implication part of the article is also somehow revised. Moreover, numerous political crises that shows the vicious cycle of identity-based conflict have been added to the article.
